# Duplicated RGS (Regulator of G-protein signaling) proteins exhibit conserved biochemical but differential transcriptional regulation of heterotrimeric G-protein signaling in *Brassica* species

**DOI:** 10.1038/s41598-018-20500-3

**Published:** 2018-02-01

**Authors:** Roshan Kumar, Naveen C. Bisht

**Affiliations:** National Institute of Plant Genome Research, Aruna Asaf Ali Marg, New Delhi, 110067 India

## Abstract

G-alpha (Gα) and ‘Regulator of G-protein Signaling (RGS)’ proteins are the two key components primarily involved in regulation of heterotrimeric G-proteins signaling across phyla. Unlike *Arabidopsis thaliana*, our knowledge about G-protein regulation in polyploid *Brassica* species is sparse. In this study, we identified one *Gα* and two *RGS* genes each from three species of *Brassica* ‘U’ triangle and assessed the effects of whole genome triplication on the divergence of gene sequence and structure, protein-protein interaction, biochemical activities, and gene expression. Sequence and phylogenetic analysis revealed that the deduced Gα and RGS proteins are evolutionarily conserved across *Brassica* species. The duplicated RGS proteins of each *Brassica* species interacted with their cognate Gα but displayed varying levels of interaction strength. The Gα and the duplicated RGS proteins of *Brassica* species exhibited highly conserved G-protein activities when tested under *in-vitro* conditions. Expression analysis of the *B. rapa RGS* genes revealed a high degree of transcriptional differentiation across the tested tissue types and in response to various elicitors, particularly under D-glucose, salt and phytohormone treatments. Taken together, our results suggest that the RGS-mediated regulation of G-protein signaling in *Brassica* species is predominantly governed by stage and condition-specific expression differentiation of the duplicated *RGS* genes.

## Introduction

Signaling through heterotrimeric G-protein (hereafter G-protein) complexes plays a fundamental role in controlling various cellular processes both in plants and animals^1,2^. The core G-protein functional complex comprises three different components *i.e*. G-alpha (Gα), G-beta (Gβ) and G-gamma (Gγ) subunits, where only Gα subunit can bind and dissociate guanine nucleotides (GTP/GDP). In animals, binding of a ligand to G-protein coupled receptor (GPCR), stimulates its guanine exchange factor (GEF) activity which promotes the release of GDP for GTP from Gα subunit, dissociating the inactive heterotrimer into two functionally independent components *i.e*. Gα-GTP and Gβγ dimer^3,4^. These two signaling units independently interact with various effector proteins which further validate their ability to participate in numerous biological functions. The intrinsic GTPase activity of the Gα subunit hydrolyzes the bound GTP and allows GDP-Gα form to reunite with Gβγ dimer, subsequently making the heterotrimer inactive^5^. In addition, GAP activity of the ‘Regulator of G-protein Signaling (RGS)’ protein is also known to accelerate the GTP-hydrolysis of Gα subunit and deactivating the G-protein cycle^6^.

Although the core components of G-protein signaling are highly conserved across phyla, the plant and animal systems are known to have enormous diversity in their quantitative repertoire and regulation of G-protein cycle. For example, the human genome encodes >800 GPCRs, 35 RGS, 23 Gα, five Gβ, and 12 Gγ proteins, regulating a wide range of biological processes^1^. In contrast, plants, in general, contain a simple repertoire of G-protein components encoding only up to four Gα and Gβ, 10 Gγ and two RGS proteins, having no prototypical GPCR^7^. Identification of multiple members of G-protein subunits has been attributed to inherent polyploidy in the angiosperm lineage^7–10^.

In addition, the activation of plant G-protein signaling is quite contrasting to the classical G-protein paradigm present in metazoans and relies on the self-activating properties of plant Gα subunit, independent of GPCR^2^. Structural and enzyme kinetic analysis of the *Arabidopsis* AtGPA1 protein plausibly explain its GPCR independent activation^11–13^. The GDP to GTP nucleotide exchange rate on AtGPA1 is approximately 100-fold faster than its rate of GTP-hydrolysis, suggesting that the plant Gα is predominantly present in the GTP-bound form^11,14^. Since G-proteins are signaling molecules, it is important to turn-off the continuing activation state of Gα-GTP after stimulation in plants. Identification of RGS proteins was primarily an important finding in plant G-protein research. In plants, the RGS protein acts as a GTPase-activating protein (GAP), accelerating the rate-limiting GTP-hydrolysis of GTP-bound Gα and so neutralizing the fast nucleotide exchange rate^6^. Interestingly, the RGS proteins reported from the plant lineage contain an N-terminal seven transmembrane (7-TM) structure which is unique and absent in their animal counterparts^11,15^. The GAP activity of plant RGS proteins is shown to be confined to the ‘RGS-domain’ present at its C-terminal region^16^. Thus, the interplay between RGS and Gα proteins is quite important in regulating overall G-protein mediated biological processes in plants. Although the plant G-protein cycle is principally known to be controlled at the deactivation step through RGS proteins; in recent years phosphorylation-dependent regulation of the G-protein cycle involving receptor-like kinases (RLKs) and their associated kinases has also been reported^17–22^.

The limited G-proteins repertoire in plants can yet control wide range of biological processes encompassing plant morphology and architecture, defence responses, abiotic stress response, sugar and phytohormone response, and yield related traits^23–34^. Although structurally similar across plant lineage, both Gα and RGS proteins interestingly possess distinct and species-specific functions. For example, the Gα mutation led to the dwarfing phenotype in rice d1 mutant^24,35^ and maize ct2 mutant^17^, whereas the *Arabidopsis* mutant (*gpa1*) did not show any significant change in plant height^36^. The Gα-RGS interplay is also known to regulate species-specific traits in plants, such as nodulation in soybean^37^. Species-specific roles of Gα and RGS proteins in plant lineage could be attributed to their distinct transcriptional and biochemical properties, as well as the involvement of their upstream regulators and downstream effectors.

*Brassica* species play an important role in global agriculture and horticulture, and share a close relationship to the model plant *A. thaliana*. The cultivable diploids (*Brassica rapa*, *B. nigra* and *B. oleracea*) and their natural allotetraploid (*B. juncea*, *B. napus* and *B. carinata*) species belonging to *Brassica* ‘U’ triangle have been well studied for their several agronomical traits like seed-yield, oil-quality, phyto-remediation, secondary metabolites, resistance against pests and pathogens^38^. The *Brassica* species are known to possess enormous genome complexity and diverse morpho-types, shaped by lineage-specific whole genome triplication (WGT) event, allopolyploidization and genomic rearrangements^39–41^. As a result, the so-called diploid *Brassica* species are paleohexaploid containing three sub-genomes and possess multiple gene homologs having variable gene expression patterns, gene-silencing effects, and neo- and sub-functionalization^42^. Although complex networks of G-protein signaling have been recently reported in few *Brassica* species^10,43^, detailed studies on the expression, biochemical, interaction and functional variance of the Gα and RGS proteins, arising from polyploidy, are fundamentally important for a better understanding of the regulation of G-protein signaling from globally cultivated *Brassica* crops.

To study the RGS-mediated regulation of G-protein signaling in *Brassica* genus, isolation of the full-length coding DNA sequence of *Gα* and *RGS* genes from three divergent species belonging to ‘*Brassica* U-triangle’ was carried out. Subsequently, the GTP-binding/hydrolysis activities of Gα orthologs; GAP activity of *Brassica* RGS proteins on Gα; and interaction selectivity between Gα and RGS proteins was examined. Later, in-depth expression profiling of *RGS* genes in various tissue types, plant developmental stages and environmental stress conditions in the *Brassica* model genome, *B. rapa* was also investigated. This work suggests that the RGS-mediated regulation of G-protein signaling in *Brassica* species is highly complex and predominantly governed by stage and condition-specific expression differentiation of duplicated *RGS* genes to control diverse growth and development processes.

## Results

### Identification and sequence analysis of *RGS* and *Gα* subunit genes from diploid *Brassica* species

**Figure 1:**
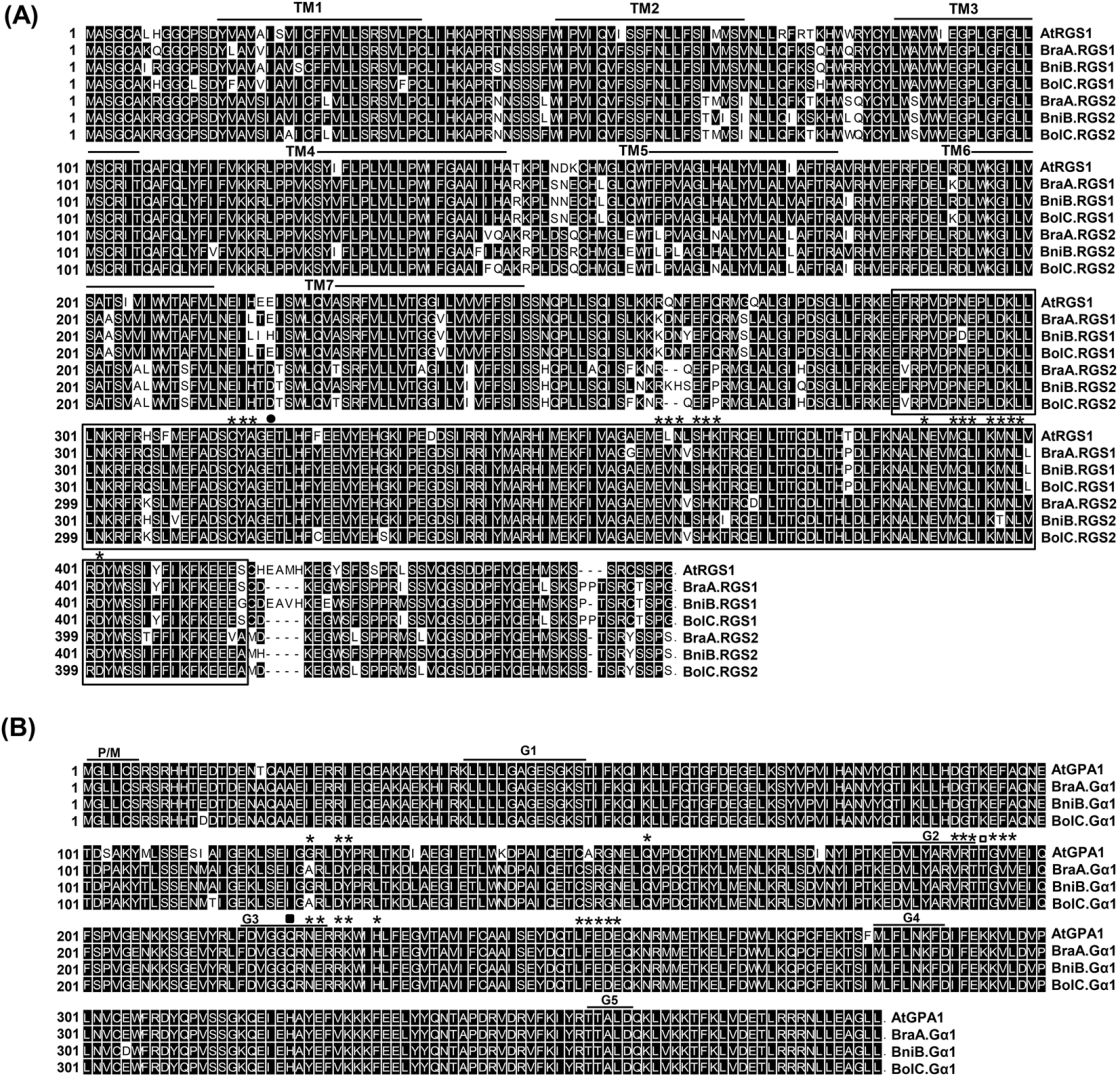
Multiple sequence alignment of *Brassica* RGS and Gα proteins. (**A**) Amino acid sequence alignment of RGS proteins from *B. rapa* (BraA), *B. nigra* (BniB*)*, *B. oleracea* (BolC) and *Arabidopsis* (AtRGS1) was performed using ClustalW (http://www.clustal.org). The predicted 7-TM domains are marked within the horizontal lines and RGS-domain is shown within the box. The critical Glu (E) residue for GAP activity of RGS protein is indicated with a filled circle. (**B**) Amino acid sequence alignment of Gα proteins from *B. rapa*, *B. nigra*, *B. oleracea* and *Arabidopsis* (AtGPA1). Position of consensus regions for GTP-binding and GTP-hydrolysis are marked within black horizontal lines (G1–G5); P/M, the predicted site for palmitoylation/myristoylation (MGXXCS); open square shows the important Thr (T) residue for RGS-Gα interaction; and the Gln (Q) residue for GTPase activity of Gα proteins is marked as filled square. The asterisks represent the important contact sites at RGS and Gα interfaces.

The full-length coding DNA sequences (CDS) of *RGS* genes from *B. rapa* (A genome), *B. nigra* (B) and *B. oleracea* (C) were amplified using the degenerate primers (Table S1). Two CDS for *RGS* genes from each *Brassica* species were isolated and designated as *BraA.RGS1* and *BraA.RGS2* (*B. rapa*); *BniB.RGS1* and *BniB.RGS2* (*B. nigra*); *BolC.RGS1* and *BolC.RGS2* (*B. oleracea*), based on the standardized nomenclature adopted for *Brassica* genus^44^. Full length coding *RGS* sequences isolated from different *Brassica* species ranged from 1368 to 1386 bp, encoding proteins of 455 to 461 amino acids in length, with an estimated molecular weight of approximately 52 kDa (Table S2). Deduced RGS proteins of *B. rapa*, *B. nigra* and *B. oleracea* shared 83.8–89.3% identity with the *Arabidopsis* AtRGS1 (Table S2). Nucleotide sequences of *Brassica RGS* genes showed 87.6–89.1% identity with *AtRGS1* (Fig. S1, Table S3). Sequence analysis of the deduced RGS proteins of *Brassica* lineage on TMHMM server (http://www.cbs.dtu.dk/services/TMHMM/) revealed the presence of an N-terminal ‘seven trans-membrane domain (7-TM)’ and a C-terminal located ‘cytosolic RGS-domain’ (Fig. 1A), similar to that reported for the *Arabidopsis* and soybean RGS proteins^15,45^. The Glu320 residue of AtRGS1 protein necessary for the GAP activity was highly conserved in all the *Brassica* RGS proteins^11^. Moreover, most of the amino acid residues recently described for the plant Gα-RGS contact interface were also found to be conserved^46^ (Fig. 1A). For example, corresponding sites for Cys316, Try317, Ala318, Glu361, Asp363, Ser365, His366, Lys367, Asp389, Met392, Gln393, Leu394, Lys396, Asp398, Leu399 and Asp402 of AtRGS1, were all found to be highly conserved across the deduced RGS proteins of *Brassica* species. Interestingly, *Brassica* lineage-specific substitution was observed at the 362^nd^ amino acid position, wherein Leu was replaced by Val. In addition, a Met397Thr substitution was also observed for the BniB.RGS2, localized in the Gα-RGS contact interface^46^.

Earlier, we reported single Gα homolog of AtGPA1 from *B. rapa*^10^ and *B. nigra*^43^ genomes. Likewise, in this study, only one Gα homolog (*BolC.Gα1*) was identified and isolated from *B. oleracea*. The pairwise sequence alignment of *BolC.Gα1* showed high sequence identity with *AtGPA1* both at nucleotide (91.8%) and protein (96.4%) levels (Fig. 1B, Table S2, Fig. S2 and Table S4). Amino acid sequence analysis of Gα proteins isolated from three diploid *Brassica* species showed conservation of characteristics guanine nucleotide binding and hydrolysis domains (G1–G5). In addition, N-terminal palmitoylation and myristoylation sites (MGXXCS) required for plasma membrane anchoring, amino acid residue for GTPase activity of Gα protein (Gln222), and most of the contact sites including Thr194 essential for Gα-RGS interaction as described by Temple and Jones^47^ and Hackenberg *et al*. (2016)^46^ were also found to be conserved in all the *Brassica* Gα orthologs (Fig. 1B).

### Evolutionary analysis of *Brassica RGS* and *Gα* genes

**Figure 2:**
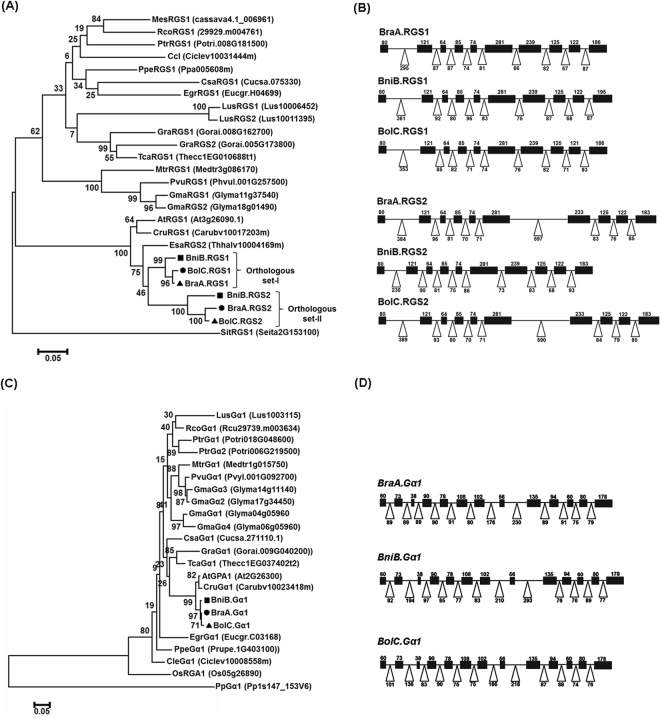
Evolutionary relationship of *Brassica* RGS and Gα proteins. The phylogenetic analysis of *B. rapa, B. nigra and B. oleracea* (**A**) RGS and (**C**) Gα proteins with other eudicots species was performed using maximum likelihood method in MEGA5.1. The names of RGS and Gα proteins used for the phylogenetic analysis are abbreviated followed by their locus ID which includes *A. thaliana* (At), *B. rapa* (BraA), *B. nigra* (BniB), *B. oleracea* (BolC), *Capsella rubella* (Cru), *Cucumis sativus* (Csa), *Citrus clementine* (Ccl), *Eucalyptus grandis* (Egr), *Glycine max* (Gma), *Gossypium raimondii* (Gra), *Linum usitatissimum* (Lus), *Manihot esculenta* (Mes), *Medicago truncatula* (Mtr), *Oryza sativa* (Os), *Phaseolus vulgaris* (Pvu), *Physcomitrella patens* (Ppe), *Populus trichocarpa* (Ptr), *Prunus persica* (Ppe), *Ricinus communis* (Rco), *Setaria italica* (Sit), and *Theobroma cacao* (Tca). The percentage of replicate trees in which the associated proteins clustered together in the bootstrap test (1000 replicates) is shown next to the branches. Gene organization of (**B**) *RGS* and (**D**) *Gα* genes in *Brassica* species. The full-length genomic sequences of *B. rapa*, *B. nigra* and *B. oleracea RGS* and *Gα* genes were retrieved from BRAD (http://www.brassicadb.org/) and phytozome (https://phytozome.jgi.doe.gov/pz/portal.html) databases. The exons and introns are shows in black box and open triangle, respectively. Number denotes the length (in bp) of respective exon and intron, drawn to scale.

A high level of amino-acid sequence identity and domain conservation of *Brassica-*specific RGS and Gα proteins led us to investigate their evolutionary relationship with the sequences reported from other plant genomes. Phylogenetic analysis showed that all RGS and Gα proteins belonging to *Brassicaceae* family were clustered together with AtRGS1 and AtGPA1, respectively (Fig. 2A,C). Interestingly, the RGS sequences identified in this study were separated into two distinct clades, named as orthologous set-I (RGS1) and set-II (RGS2), suggesting duplication of *RGS* genes in *Brassica* lineage. The RGS proteins belonging to orthologous set-I were evolutionarily closer to the AtRGS1. Further, within each orthologous set, the RGS proteins from *B. rapa* and *B. oleracea* showed a close phylogenetic relationship, compared to its *B. nigra* counterpart. Comparison of synonymous substitution rate (Ks) value between the duplicated *RGS* genes isolated from each *Brassica* genome showed that *RGS1* orthologs have lower Ks (0.34–0.36) values than *RGS2* orthologs (0.41–0.47), signifying differential divergence of the duplicated RGS proteins (Table S5). Divergence time analysis showed that the duplicated *RGS1* and *RGS2* genes of each *Brassica* species diverged around 11.62–15.68 mya, very soon after the *Arabidopsis-Brassica* split event, estimated around 13–17 mya^40^. Interestingly, our data revealed that the duplicated *RGS* genes of *B. nigra* have higher Ks values compared to their *B. rapa* and *B. oleracea* counterparts. The Gα sequences identified in all the three *Brassica* species shared highly similar Ks values (0.39–0.42), estimated to diverge around 13.07–14.23 mya from the *AtGPA1* (Table S5).

Based on the *RGS* and *Gα* coding sequences isolated in this study, we retrieved their genomic counterparts from the recently assembled *Brassica* database (http://brassicadb.org/brad/). The *BraA.RGS1* (corresponds to Bra025181), *BraA.RGS2* (Bra017336) and *BraA.Gα1* (Bra007761) were localized onto A06, A09, and A09 chromosomes in the model *B. rapa* (A) genome (Table S6). Similarly, the *RGS* and *Gα* orthologs from *Brassica* ‘B’ (*B. nigra*) and ‘C’ (*B. oleracea*) were also identified and summarized in Table S6. Gene structure analysis revealed that the *RGS* genes contain 10 exons and 9 introns, whereas the *Gα* genes harbour 13 exons and 12 introns (Fig. 2B,D). Intron-exon organization of the G-protein orthologs present in *B. rapa* and *B. oleracea* was quite similar compared to their *B. nigra* counterparts, where the size of introns was found to be variable.

**Figure 3:**
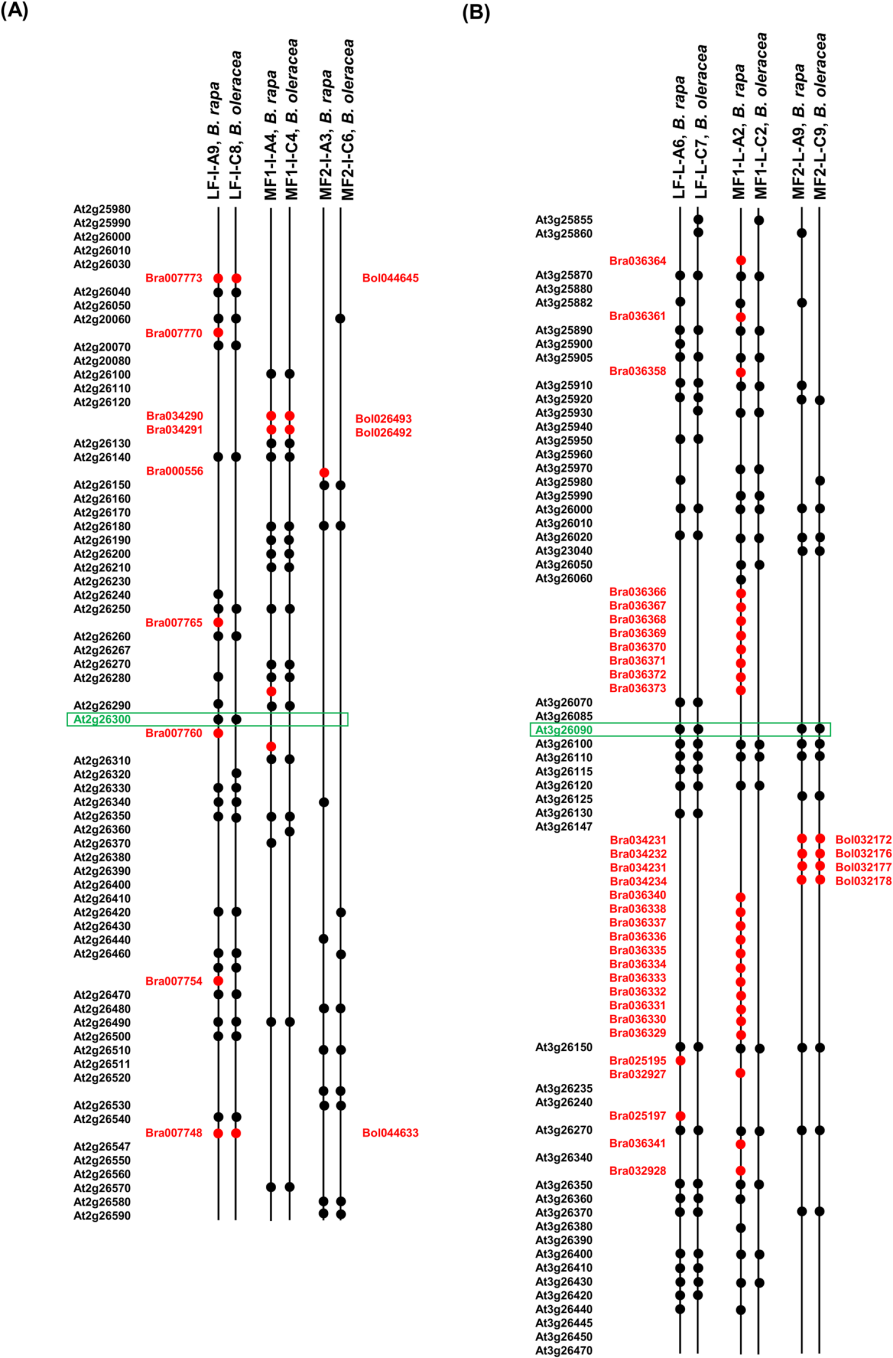
Comparison of gene organization in the genomic blocks ‘I’ and ‘L’ of *A. thaliana*, *B. rapa* and *B. oleracea*, as obtained from BRAD database (http://www.brassicadb.org/). Gene arrangement of *B. rapa* and *B. oleracea* syntenic orthologs of 25–30 representative *A. thaliana* genes flanking each side of (**A**) *AtGPA1* within genomic block ‘I’, and (**B**) *AtRGS1* within genomic block ‘L’. The syntenic position of *AtGPA1* (At2g26300) and *AtRGS1* (At3g26090) genes are marked within green boxes in (**A**) and (**B**), respectively. Syntenic genes shared between the three species are represented with black circle; *Brassica* lineage specific genes are marked with red circle showing their gene ID. The line nomenclature describes the ‘subgenome-genomic block-linkage group-*Brassica* species’.

*Brassica* species are mesohexaploids, containing three sub-genomes (LF, MF1 and MF2) formed due to the whole-genome-triplication event^48^. Sub-genomic distribution analysis of the G-protein candidate genes in *B. rapa* and *B. oleracea* genomes (http://brassicadb.org/brad/) revealed that the *Gα* and *RGS1* orthologs were present on LF (least fractionized) sub-genome, whereas *RGS2* was present on MF2 (most fractionized) sub-genome (Fig. 3). Homologs of *RGS* and *Gα* genes were occupied within the *Brassica* ancestor genomic block ‘L’ and ‘I’, respectively. To get evolutionary insight into this differential gene-retention, we further analyzed the gene content within the genomic blocks ‘L’ and ‘I’ shared between *A. thaliana* and the three sub-genomes of sequenced *B. rapa* and *B. oleracea* genomes (Table S7). The gene-retention frequencies of ‘L’ and ‘I’ genomic blocks belonging to LF sub-genome were almost similar (42.1–47.0%), whereas within the MF2 sub-genome the gene retention of genomic block ‘L’ (19.8–20.7%) was comparably higher than genomic block ‘I’ (10.3–10.5%), thereby suggesting uneven gene-fractionation of the triplicated sub-genomes in *Brassica* species.

### Protein-protein interaction between duplicated RGS and Gα proteins of *Brassica* species

**Figure 4:**
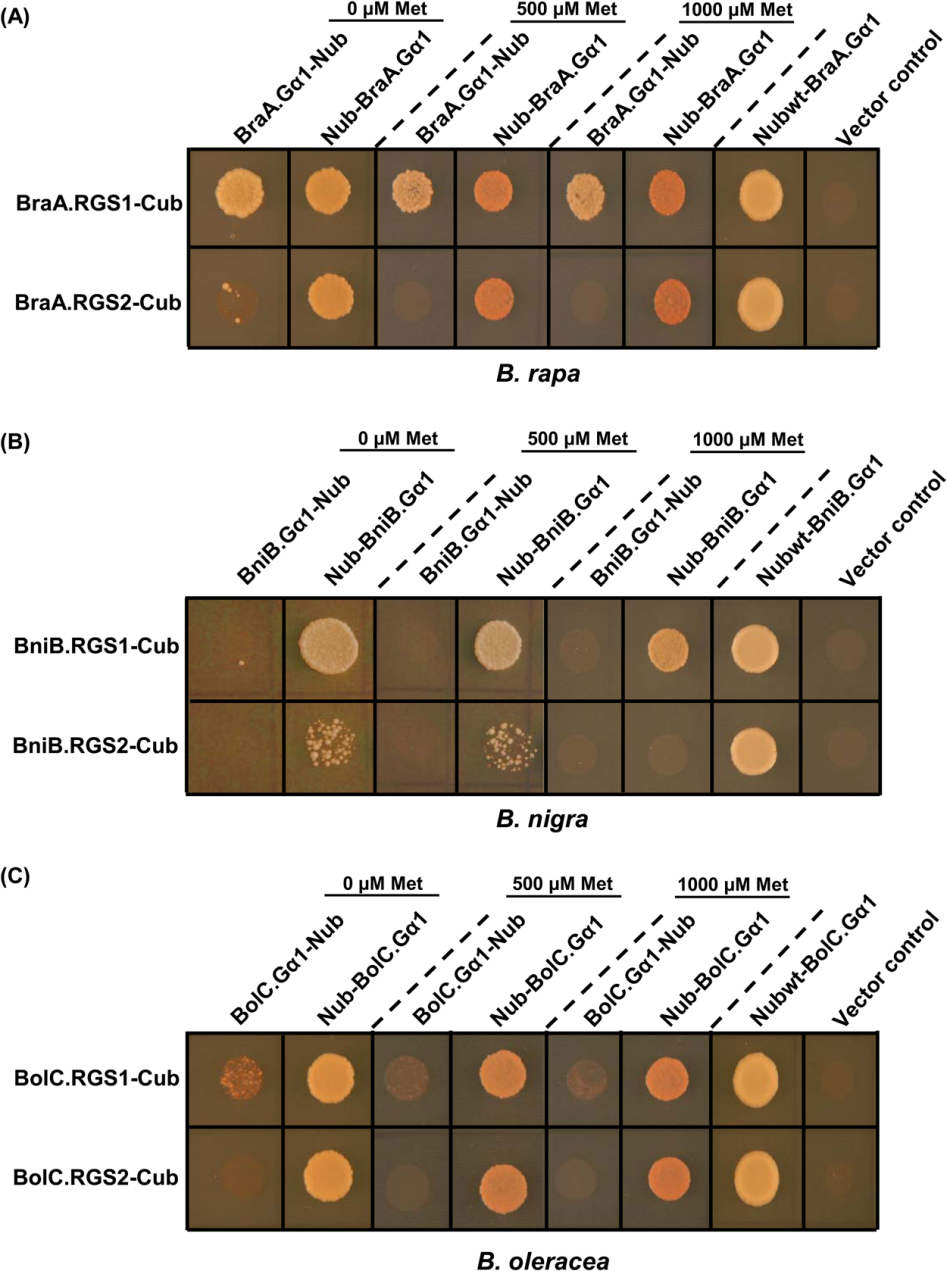
Interaction between Gα and RGS proteins of *Brassica* species namely (**A**) *B. rapa*, (**B**) *B. nigra*, and (**C**) *B. oleracea* using mating based split ubiquitin system. Gα subunit was cloned in both the orientation of Nub vector (containing N-terminal half of ubiquitin), and RGS proteins were cloned in Cub vector (C-terminal half of ubiquitin), as fusion proteins. The interaction was examined by the growth of mated diploid yeast cells on selection plates (SD-AHLT) containing 0, 500 and 1000 μM Met. The NubWt-Gα protein and empty NubG vector were used as positive and negative control, respectively. Two biological replicates of the experiment were performed with identical results.

In metazoans, where multiple members of RGS and Gα subunit are present, interaction specificity between various Gα-RGS proteins controls the kinetics of nucleotide (GTP/GDP) cycling and sensitivity of G-protein signaling^49^. In plants, studying the Gα-RGS interaction is quite important considering that Gα protein has self-activating property and so far RGS and PLDα1 are the only well-studied modulators of G-protein signaling^50,51^. In this study, two divergent RGS proteins were identified each from the three diploid *Brassica* genomes. To analyse the interaction strength and specificity between the duplicated RGS proteins and Gα subunit, a mating based split ubiquitin system (mbSUS) assay was performed, wherein the Gα and RGS proteins were used as prey and bait proteins, respectively. Based on the growth of mated yeast cells on selection medium, we observed that duplicated RGS proteins of each *Brassica* species interacted with their cognate Gα protein (Fig. 4). In *B. rapa* and *B. oleracea*, RGS1 protein showed strong interaction with their respective Gα subunit in both the orientations, while RGS2 protein could interact only in one orientation of Gα (as a C-terminal fusion of Nub, Nub-Gα) when tested on three different concentrations of Met (Fig. 4A,C). The duplicated RGS proteins of *B. nigra* interacted with its cognate BniB.Gα1 specifically in one orientation (Nub-Gα), wherein the BniB.RGS2 showed relatively weak interaction (Fig. 4B).

In Arabidopsis, the ‘RGS-domain’ present at the C-terminal region of RGS protein is known to interact with the AtGPA1^16^. Therefore, to further validate our observation, we analysed the interaction of the C-terminal cytosolic domain containing the RGS-box of the duplicated RGS proteins of *Brassica* origin with their cognate Gα protein by utilizing the conventional GAL4 based yeast two hybrid (Y2H) system. The RGS-domains of duplicated RGS proteins of *B. rapa* showed strong and comparable interaction with BraA.Gα1 even up to 25 mM of 3AT (Fig. S3). However, as also observed for the full-length RGS proteins, the RGS-domains of the two *B. nigra* proteins showed differential interaction specificity with BniB.Gα1. The RGS-domain of the BniB.RGS1 showed strong interaction with BniB.Gα1, compared to BniB.RGS2 showing weak interaction. Hence, data obtained from both the mbSUS and Y2H assays, clearly suggest that duplicated RGS proteins interacted with their cognate Gα subunit in *Brassica* species, although showing a varying level of interaction specificity.

### GTP-binding/hydrolysis activity of Gα protein and GAP activity of duplicated RGS proteins in *Brassica* species

**Figure 5:**
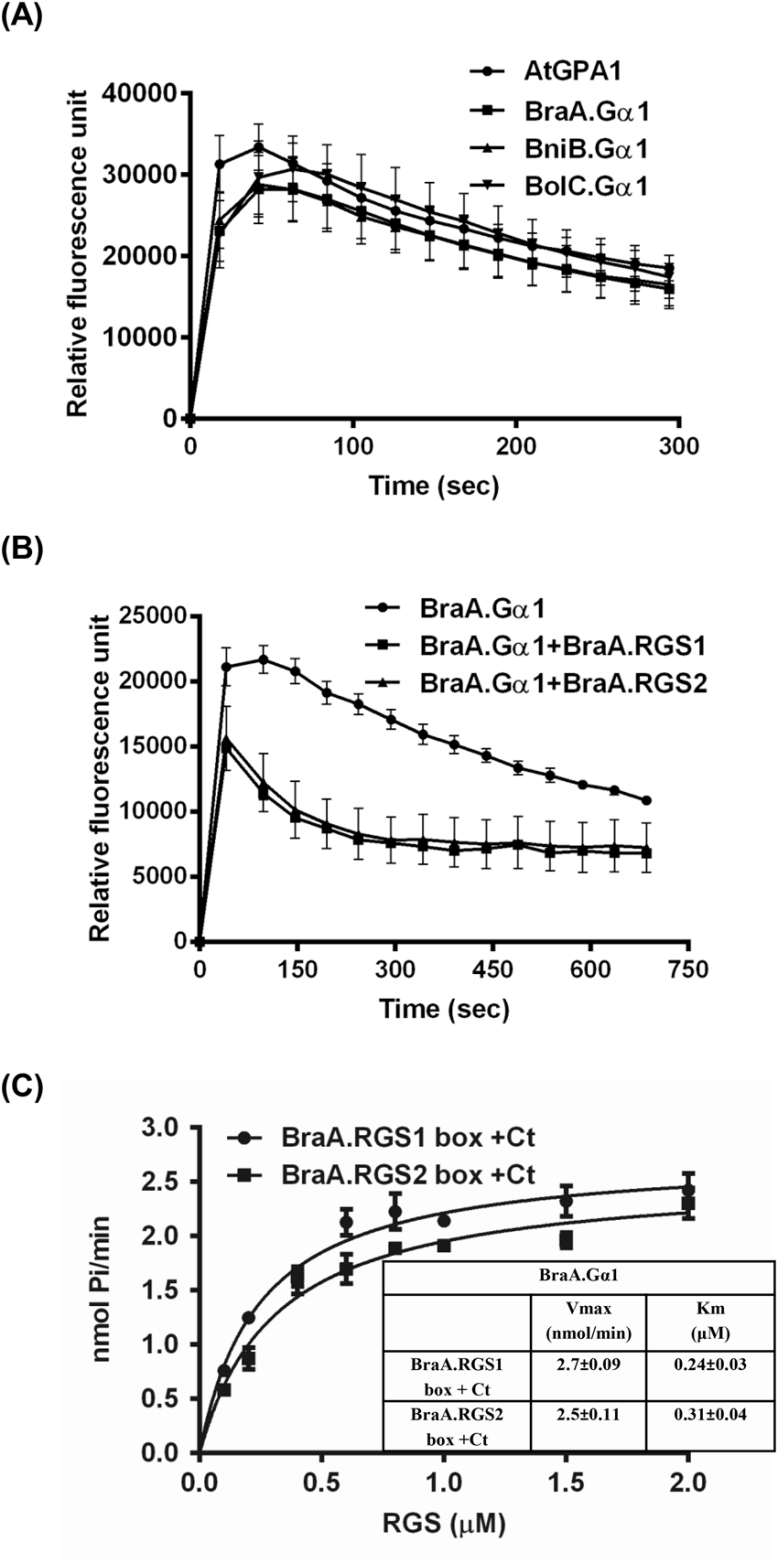
Biochemical characterization of *Brassica* Gα and RGS proteins. (**A**) GTP-binding/hydrolysis activity of the recombinant Gα protein using BODIPY fluorescent dye in real-time fluorescence assays; (**B**) Effect of the recombinant BraA.RGS1 and BraA.RGS2 domains (expressed as C-terminal region containing RGS-box) on GTP-hydrolysis of BraA.Gα1 using BODIPY fluorescent dye in real-time fluorescence assays; and (**C**) *In-vitro* Pi release activity of BraA.Gα1 in the presence of different concentration of BraA.RGS1 and BraA.RGS2 domains. Inset table shows the kinetic parameters of Pi release from GTP-bound Gα protein, in the presence of RGS domains of the duplicated *B. rapa* RGS proteins. Experiments were carried out three times and data was averaged. Error bars represent the mean (±)SE. Data were analyzed using GraphPad Prism version 6.0.

Among the different G-protein core components, the biochemical properties of Gα subunit are contrasting between plants and animals thereby making the paradigm of G-protein signaling quite interesting. In order to get a primary insight into the regulation of G-protein in *Brassica* species, recombinant Gα proteins containing N-terminal His-tag were purified using Ni-NTA affinity chromatography (Fig. S4A) and an *in-vitro* activity assay of recombinant Gα proteins was carried out using BODIPY-GTP FL fluorescent dye in a real time fluorescent assay. The rates of GTP-binding (increase in fluorescence) and GTP-hydrolysis (decrease in fluorescence) of all the three *Brassica* Gα proteins was found to be highly comparable, and similar to that observed for the *Arabidopsis* AtGPA1 (Fig. 5A). In general, the intrinsic GTP-hydrolysis activity of the *Brassica* Gα proteins was found to be very slow.

Slow GTP-hydrolysis activities of Gα proteins indicate the important role of RGS proteins in regulating G-protein cycle in genus *Brassica*. Due to a high level of sequence similarity between RGS1 and RGS2 orthologs across *Brassica* species, we initially selected duplicated RGS proteins of the model *Brassica* genome, *B. rapa*, and for biochemical characterization of the same. The C-terminal region containing RGS-domain of the *B. rapa* RGS proteins *i.e*. BraA.RGS1-box (296–417 amino acids) and BraA.RGS2-box (284–415 amino acids) containing N-terminal His-tag were heterogeneously expressed in *E. coli* and purified using Ni-NTA based affinity chromatography (Fig. S4B). Real-time assays using BODIPY-GTP FL showed that both BraA.RGS1 and BraA.RGS2 accelerated the GTP-hydrolysis of their cognate BraA.Gα1, and the GTPase (GAP) activity was found to be somewhat similar for both of the duplicated BraA.RGS1/2 proteins (Fig. 5B). Likewise, the GAP activity of the duplicated RGS proteins of *B. nigra* and *B. oleracea* also showed similar trend on their cognate Gα proteins (Fig. S5A,B). Further, to determine an accurate rate of GAP activity of the duplicated BraA.RGS proteins, steady state kinetics of Pi release was carried out using 1 μM of BraA.Gα1 and 0.1–2.0 μM of BraA.RGS proteins. BraA.Gα1 showed a marginal difference in its rate of Pi release when tested using the BraA.RGS1 (Km 0.244 ± 0.03; Vmax 2.70 ± 0.09) and BraA.RGS2 (Km 0.311 ± 0.04; Vmax 2.55 ± 0.11) proteins (Fig. 5C). Overall, the presence of highly similar GTP-binding/hydrolysis activities of Gα proteins, and somewhat similar GAP activities of the duplicated RGS proteins, in all possibility, suggest that the RGS-mediated G-protein regulation is biochemically conserved in *Brassica* lineage.

### Transcript expression and sub-cellular localization of *B. rapa* duplicated RGS proteins

**Figure 6:**
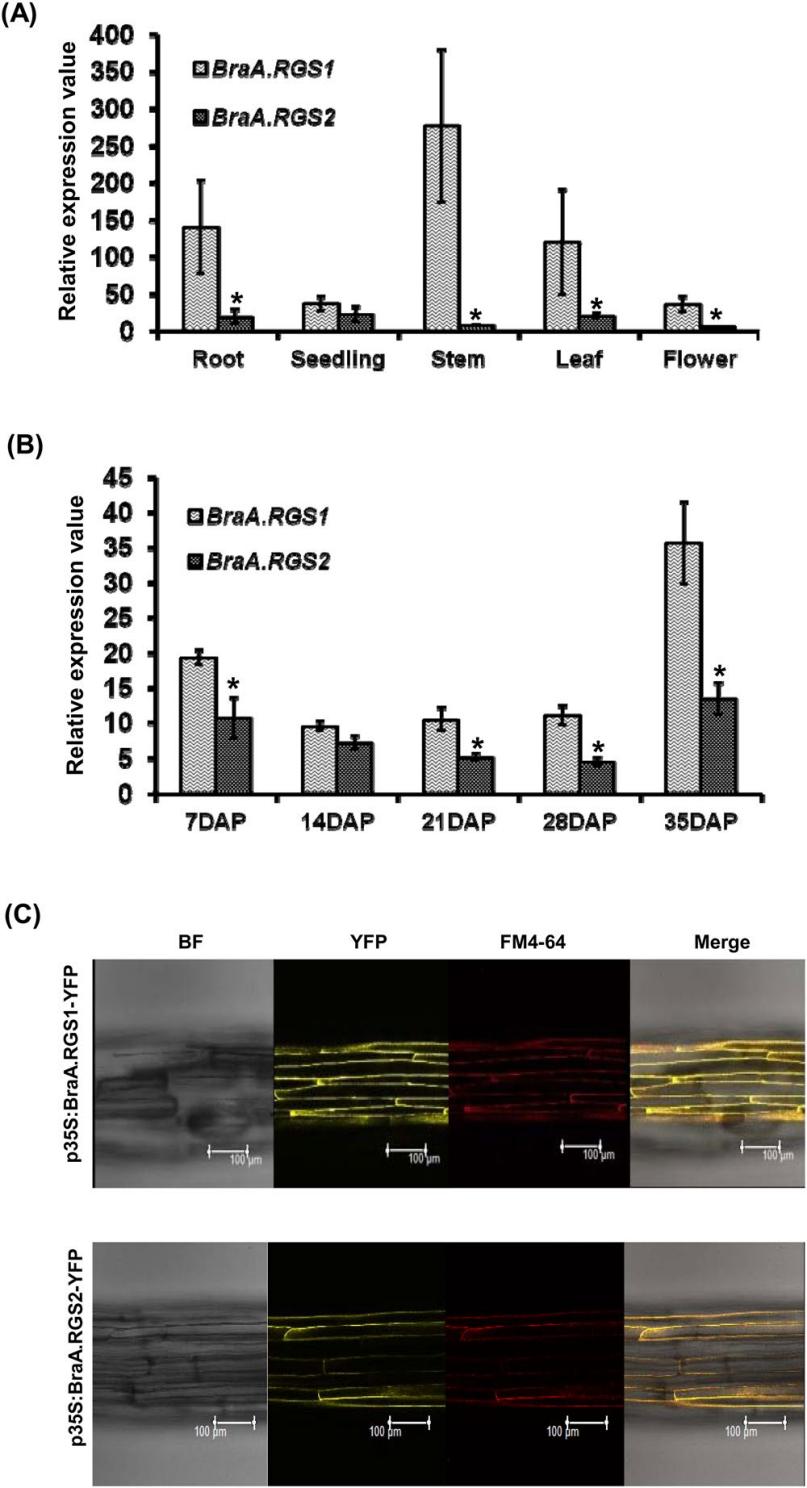
Transcript expression profiling of the duplicated *RGS* genes in (**A**) different tissue types, and (**B**) seed maturation stages of *B. rapa*. Real time PCR amplifications were performed for each target gene in three biological replicates with two technical replicates each. The expression of *TIPS-41* for different tissue types and *GAPDH* for seed maturation stages were used to normalize the data (set at 100). Error bars represent the standard error. Significant expression differences were calculated at P < 0.05 using independent sample t-test using SPSS statistic version 17 software and marked with asterisk on the top of the error bars. (**C**) Sub-cellular localization of BraA.RGS1 and BraA.RGS2 proteins. Four days old *Arabidopsis* seedlings (dark grown) showing localization of BraA.RGS1-YFP and BraA.RGS2-YFP fusion proteins (yellow) in plasma membrane of hypocotyl cells. FM4-64 (red) was used to stain the plasma membrane, and merged images are shown in the right column.

Further, to investigate the transcriptional regulation of the duplicated *RGS* genes, we carried out gene expression analysis in the model *Brassica* species, *B. rapa*. Real-time qRT-PCR analysis revealed that both the *B. rapa RGS* genes (paralogs), resulting from WGT event, were expressed, showing a contrasting difference in their expression patterns. In general, *BraA.RGS1* had higher expression compared to *BraA.RGS2* in most of the tissue types, except seedlings (Fig. 6A). The expression of *BraA.RGS1* was also found to be higher than *BraA.RGS2* during all stages of seed development (Fig. 6B). Interestingly, the expression of both *BraA.RGS1* and *BraA.RGS2* was found to be up-regulated during later stages of seed maturation (35 dap, days-after-pollination) suggesting their important during seed development in *B. rapa*. Sub-cellular localization studies in transgenic *Arabidopsis* hypocotyls revealed that the C-terminal YFP-fusion of both BraA.RGS1 and BraA.RGS2 proteins were localized in the plasma-membrane, along with FM4-64 (red), a dye used for staining cell membrane (Fig. 6C). Moreover, transient expression studies in *N. benthamiana* epidermal cells also established that the duplicated RGS proteins of *B. rapa* are localized in the plasma-membrane (Fig. S6), as also reported for the soybean RGS proteins^45^.

### Expression analysis of *B. rapa* duplicated *RGS* genes under various elicitor treatments

**Figure 7:**
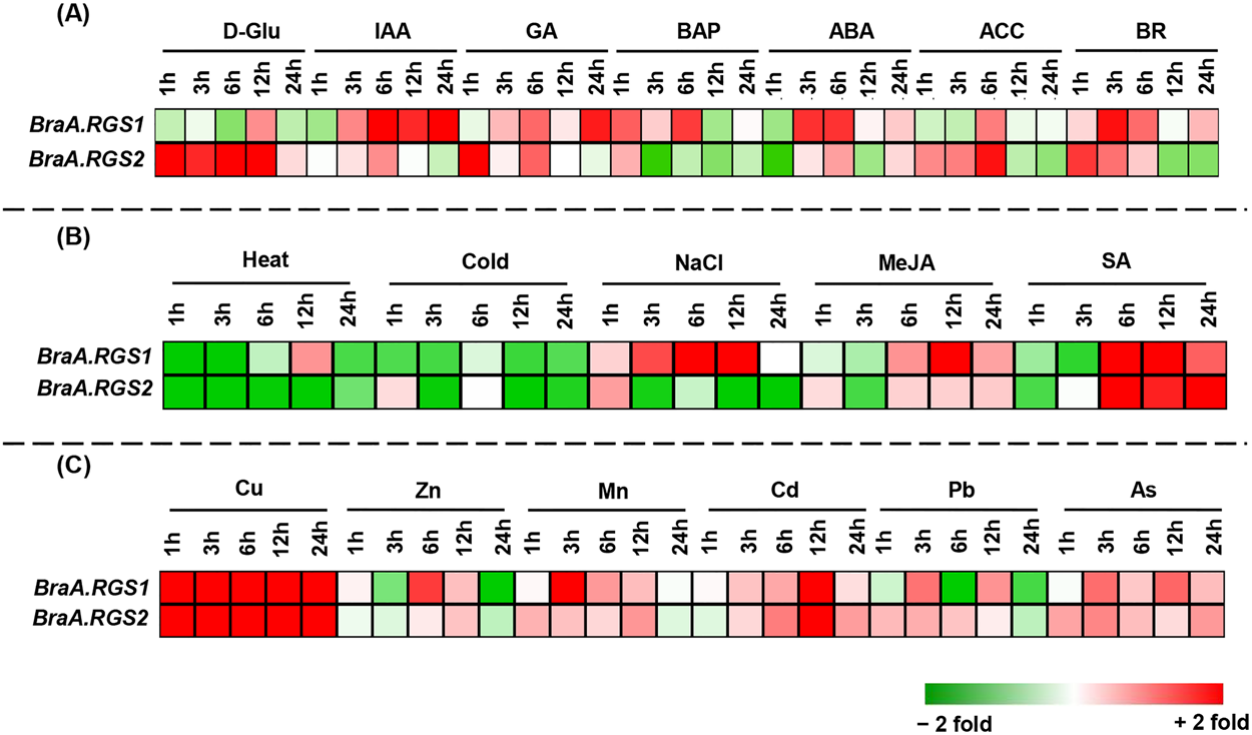
Expression pattern of the duplicated *RGS* genes under various elicitor treatments in *B. rapa*. Heat map of genes showing the effects of (**A**) D-Glucose and phytohormones; (**B**) abiotic and biotic stress conditions; and (**C**) heavy metals on transcript level of *RGS* genes in *B. rapa*. Real-time PCR analysis was conducted at five time points (1, 3, 6, 12 and 24 h) of treatments, and the data was averaged (n = 4) and normalized using of *Ubiquitin* (*UBQ*) gene expression. The colors on heat map represent the up-regulation (≥2 fold, red) and down-regulation (≤2 fold, green) of the *RGS* transcripts compared with the untreated mock seedlings grown in liquid 0.5X MS medium for similar time points.

So far, information about the roles and regulation of plant *RGS* genes under various developmental and environmental cues is sparse and mostly limited to the model plant *A. thaliana*. To get an initial insight into the transcription regulation of the duplicated *RGS* genes, in-depth transcript expression profiling was carried out in *B. rapa*. Five-day-old uniform seedlings of *B. rapa*, grown on 0.5 × Murashige and Skoog (MS) medium containing 3% sucrose, were subjected to different elicitor treatments, including D-glucose, phytohormones, abiotic and biotic stress conditions for 1, 3, 6, 12 and 24 hours (h) as described previously^43,52^. qRT-PCR analysis showed a significant up-regulation of *BraA.RGS2* transcript compared to *BraA.RGS1* when treated with D-glucose for all the tested time points (Fig. 7A), thereby suggesting a differential transcriptional response of the *B. rapa* duplicated *RGS* genes under glucose treatment. The *BraA.RGS* genes also showed differential expression patterns in response to exogenously supplied phytohormones. Expression of *BraA.RGS1* transcript was, in general, found to be up-regulated during most of the tested time points of phytohormone treatments, including IAA, GA, BAP, ABA and BR (Fig. 7A). However, the transcript abundance of *BraA.RGS2* was found to be up-regulated only during early time points for most of the phytohormone treatments. The duplicated *RGS* genes also showed distinct expression patterns in response to various abiotic and biotic stress conditions. A profound up-regulation of both *BraA.RGS1* and *BraA.RGS2* was observed under SA treatment, whereas these transcripts showed significant down-regulation under heat and cold treatments (Fig. 7B). Further, differential transcriptional regulation of the duplicated *RGS* genes was also observed in response to both NaCl and MeJA treatments, wherein the *BraA.RGS1* transcript showed a significant up-regulation upon these treatments.

*Brassica* species are known to be the heavy metal accumulators and are globally used for phyto-remediation purposes. We, therefore, studied the expression patterns of the duplicated *RGS* genes under various heavy-metal ion toxicity in *B. rapa*. Heavy metal treatment showed substantial parallelism in the expression patterns of the duplicated *RGS* genes (Fig. 7C). Interestingly, we found a high accumulation of *BraA.RGS1* and *BraA.RGS2* transcript under Cu stress during all the tested time points (1 to 24 h). Likewise, up-regulation of the duplicated *RGS* genes was also observed under Cd, Mn and As stress although, showing a time-dependent transcript accumulation. Under Zn and Pb treatments, we found differential transcript responses of the duplicated *RGS* genes during different time points. The profound up-regulation of the duplicated *RGS* genes in all possibility suggests their key involvement during heavy-metal ion toxicity in *Brassica* crops.

## Discussion

Over the recent years, it has become increasingly clear that plants have a unique mechanism of G-protein signaling, owing to limited repertoire of core G-protein components, fast GTP-binding with very slow intrinsic GTPase activity of Gα proteins, and most importantly the absence of functional GPCRs in plants. Among the various G-protein signaling components reported across the plant lineage, the physical interaction and biochemical activities of Gα and RGS proteins are quite crucial for regulating the G-protein cycle^53^.

### *Brassica* genomes encode highly conserved Gα and RGS proteins

Various comparative genomics and genome sequence studies have unequivocally reported the existence of a WGT event in *Brassica* lineage, after its split from the model plant *Arabidopsis*, dating around 13–17 mya, as a result of which the so-called diploid *Brassica* species are paleohexaploid containing three sub-genomes (LF, MF1 and MF2)^39–41^. Although three copies of each of the *Arabidopsis* ortholog are quite expected, in the current study only one *Gα* and two *RGS* genes were identified in each of the three *Brassica* genomes, present in LF and MF2 sub-genomes only (Fig. 3, Table S7). The uneven expansion of the candidate G-protein genes in *Brassica* species could be attributed to the biased gene fractionation (gene-loss) frequency across the three sub-genomes^41,48^. Notably, within the sub-genome MF2, the genomic block containing *Gα* (I) encountered higher gene-loss than the *RGS* containing genomic block (L). Our observation was quite in agreement with earlier reports describing the uneven expansion of key signaling genes in *B. rapa*, particularly involved in G-protein and 14-3-3 signaling pathways^10,52^.

Sequence and phylogenetic analysis of both RGS and Gα proteins suggest that these proteins are evolutionarily conserved in *Brassica* lineage (Figs 1 and 2), wherein the Gα orthologs have significantly high sequence conservation compared to the duplicated RGS proteins. This in all possibility suggests that the canonical Gα proteins might have retained highly conserved biochemical activities vis-à-vis biological functions during the evolution of extant *Brassica* species. In such scenario, the presence of divergent RGS or other regulatory proteins could play an important role in regulating the G-protein cycle and signaling in these mesohexaploid *Brassica* species. The differential synonymous base substitution (Ks) rates observed for the duplicated RGS proteins suggests their differential functional specificity and interaction selectivity with the Gα and other effector proteins, which needs detailed investigation.

### *Brassica* Gα and RGS proteins display differential interaction specificity

Expansion of the repertoire of G-protein components revealed the presence of complex signaling network in plants^7^. These components tend to interact in various combinations to govern the functional selectivity in various cell and/or tissue types. Various studies in animal systems, clearly established the significance of RGS and Gα interactions in regulating the GAP activity^54^. Our data suggest that during evolution, the canonical Gα protein present in each *Brassica* species has retained strong interaction with the ancestral RGS1 protein compared to the recently evolved RGS2 protein (Figs 4, S3). Divergent residues present between the duplicated RGS proteins (Fig. 1) could govern this differential interaction specificity with the canonical Gα protein. Interestingly, among the RGS2 orthologs present across *Brassica* species, the *B. nigra* BniB.RGS2 showed comparatively weaker interaction with its cognate BniB.Gα1. A comparably higher Ks value of the *BniB.RGS2* and various invariant residues, preferentially present at the C-terminally located RGS-domain (including Met397Thr substitution), might alter the stability and interaction of the Gα-RGS contact interface^46^, which needs further investigation. The predominance of BniB.RGS1-BniB.Gα1 interaction in all possibility suggested that the regulation of G-protein signaling is quite distinct in *B. nigra*, and somewhat different from the *Brassica* A/B genomes. Noteworthy, the gene-structure analysis in our study also revealed that the intron-exon attributes of both *Gα* and *RGS* genes in *B. nigra* varied somewhat from their *B. rapa* and *B. oleracea* counterparts (Fig. 2). These observations could be best explained by the fact that *B. nigra* has evolved separately from the *B. rapa/B. oleracea* lineage in the tribe Brassicaceae^55^. Nonetheless, the differential RGS-Gα interaction specificity suggests distinct RGS-mediated regulation of G-protein signaling in *Brassica* lineage. Detailed functional studies using gain- and loss-of-function strategies of G-protein genes in each of these *Brassica* species need to be undertaken to uncover the significance of this differential interaction.

### The canonical Gα and duplicated RGS proteins display similar G-protein activities in *Brassica* lineage

The unusual self-activating property of Gα protein makes the plant G-protein signaling unique from that of metazoans. The plant Gα proteins posses fast rate of GTP-binding and slow GTP-hydrolysis ability, which are quite contrasting to their animal counterparts^2^. In this study, we observed that the canonical Gα orthologs of three *Brassica* species are biochemically active and display highly similar GTP-binding and GTP-hydrolysis activities, similar to the *Arabidopsis* AtGPA1 (Fig. 5). This indicates that during the evolution of extant *Brassica* species, changes in few amino acid residues do not seem to impart any significant differences on the activities of these Gα proteins, at least under the tested *in-vitro* conditions. In plants, GTP-hydrolysis of Gα is the rate limiting step of G-protein cycle and is modulated by the GAP activity of RGS proteins^11^. Our *in-vitro* data based on fluorescence assays and steady state kinetics experiments show that the cytosolic RGS-domain of the duplicated RGS proteins increases the rate of GTP-hydrolysis of the cognate Gα, and are active GAP proteins (Fig. 5). Further, comparable GAP activities of the active RGS-box of duplicated RGS proteins in *Brassica* lineage suggest that the RGS-mediated regulation of G-protein cycle in *Brassica* crops is biochemically conserved, although other modes of regulation may be expected.

Our observation is different from that reported in allotetraploid soybean genome, where the four Gα and duplicated RGS protein exhibit distinct G-protein activities^8,45^. The distinct activities of G-protein regulatory elements can be best explained by the fact that the soybean genome has experienced two rounds of whole genome duplication (WGD) events dating around 58–60 mya (ancient duplication) and a recent duplication ca. around 13 mya^56^. Since the paralogs created during the ancient WGD event are expected to diverge out considerably, the G-protein members in soybean have retained distinct G-protein activities and functional divergence to regulate various plant growth and developmental traits^37^. However, multiple homologs formed in *Brassica* lineage have resulted from a very recent WGT event (~13–15 mya), as a result of which the duplicated/triplicated genes (paralogs) could have retained comparably higher sequence identity vis-à-vis similar biochemical activities and biological functions in the extant *Brassica* species, as evident from our current study.

### Differential transcriptional regulation of duplicated *RGS* genes under plant developmental stages and elicitor treatments

Over time, the duplicated genes formed as a result of WGD and WGT events in polyploids are known to alter their gene expression, as a result of which these genes undergo different evolutionary fates including neo-functionalization, sub-functionalization and pseudogenization^57,58^. The transcriptional differentiation of multiple gene homologs is well documented in polyploid plant species^59^. In this study, the duplicated *RGS* genes of *B. rapa* showed a high degree of transcriptional bias across various developmental stages wherein *BraA.RGS1* was found to be transcriptionally more active compared to *BraA.RGS2* (Fig. 6). Considering only one canonical Gα subunit, and conserved biochemical activities of both Gα and duplicated RGS proteins present across *Brassica* species, it is quite possible that the RGS-mediated G-protein regulation could be dependent on the differential transcriptional response of duplicated *RGS* genes to regulate various assets of plant growth and development, as also proposed for soybean^45^. Moreover, in *Brassica* lineage where WGT is an inherited norm the sub-genome dominance also shapes the expression and functional dominance of paralogous gene within a gene family^42,52^. The sub-genome dominance effect is also evident in this study, wherein the highly expressed BraA.RGS1 is localized in the transcriptionally active least-fractionized (LF) sub-genome of *B. rapa*.

Both loss- and gain-of-function studies in plants under phytohormones and other elicitor treatments show the significance of G-proteins in controlling various assets of plant growth and development. The *Arabidopsis* G-protein mutants exhibit differential phenotypic response to auxin and ABA treatments. For example, *gpa1* mutant displayed hypersensitivity towards ABA during seedling and root development^60^, whereas, it showed wild-type like inhibition of seedling growth; and hyposensitivity in lateral root development to auxin treatment^25,28^. Among the two *RGS1* genes of *B. rapa*, a higher up-regulation of *BraA.RGS1* during IAA and ABA treatments is quite evident and might suggest its preferential cross-talk with auxin and ABA-mediated seedling development. The *Arabidopsis* G-protein mutants show hypersensitive response to D-glucose during early stages of plant development like seed germination, seedling development and root growth^2^. In recent years, it has been well documented that upon D-glucose and NaCl treatments, AtRGS1 undergoes endocytosis from the plasma-membrane to the endosomes which lead to physical uncoupling of AtRGS1 from AtGPA1, thereby allowing the AtGPA1 to self-activate^61,62^. Among the duplicated *RGS* genes, the up-regulation of *BraA.RGS1* and *BraA.RGS2* expression in response to NaCl and D-glucose treatment, respectively, suggest their specific roles in regulating various salt and sugar-responsive phenotypes in *B. rapa*. The involvement of G-protein components in plant defence is quite established in *Arabidopsis*^26,27,29,34^. In addition, the up-regulation of the duplicated *BraA.RGS* genes under SA treatments possibly suggests their coordinated involvements in defence signaling in *B. rapa*.

Quite interestingly, both the duplicated *RGS* genes are highly up-regulated in response to Cu and Cd treatments, suggesting their potential roles during heavy-metal toxicity. Recently, Kunihito *et al*.^63^ also showed the involvement of G-proteins in conferring Cd tolerance in yeast and *Arabidopsis*. Thus RGS-mediated G-protein signaling could represent a novel pathway for phyto-remediation of heavy-metal ions in *Brassica* species, although other, yet unknown, mechanism may also exist, which warrants further investigation.

Overall, our study shows that the transcriptional differentiation of the biochemically conserved RGS proteins could be quite important to condition-specific Gα-RGS interaction vis-à-vis biological functions in the *Brassica* lineage. A detailed characterization of *Gα* and *RGS* genes could be carried out to integrate the multiple molecular connections that co-ordinately regulate the strength and/or duration of G-protein signals in controlling the various assets of plant growth and development in the globally important *Brassica* crops.

## Materials and Methods

### Plant material and growth conditions

Three *Brassica* species namely, *B. rapa* L. (cv. YIDI), *B. nigra* L. (cv. IC257) and *B. oleracea* L. (cv. Golden Acre) used in the present study were grown under controlled growth conditions at day (24 °C; 10 h; ca. 300 μmol.m^−2^.s^−1^) and night (18 °C; 14 h) photoperiod with 55–60% relative humidity. Tissue types representing different developmental stages of *B. rapa* including five-day old seedlings, fully developed leaves, root, stem, flower and different stages of developing siliques (7 to 35 days-after-pollination) were collected and stored at −80 °C.

### Amplification and cloning of *Gα* and *RGS* CDS from *Brassica* species

The standard PCR amplification conditions were deployed with an annealing temperature of 55 °C (30 sec) to obtain the full-length coding DNA sequence of *Gα* and *RGS* genes from *B. rapa* (A genome), *B. nigra* (B genome) and *B. oleracea* (C genome). The cloning of Gα CDS from *B. rapa* and *B. nigra* has been reported in our earlier studies^10,43^, whereas from *B. oleracea* was performed in the present study. For cloning *RGS* genes, the primers were designed based on the *Arabidopsis* ortholog (*AtRGS1*) and annotated *B. rapa* genes available in the phytozome database (Locus ID: Brara.F03296.1 and Brara.I02169.1) (Table S1). Subsequently, PCR products were cloned into the pENTR/D-TOPO vector (Invitrogen, USA) and sequenced to confirm their fidelity. At least, three independent PCR amplifications were carried out to confirm the gene sequences.

### Sequence alignment, phylogenetic and divergence analysis

Phylogenetic analysis of the deduced RGS and Gα protein sequences isolated from *B. rapa*, *B. nigra*, *B. oleracea*, and those retrieved from different plant species (https://phytozome.jgi.doe.gov/pz/portal.html) was carried out using the maximum likelihood method in MEGA5.1 with 1,000 bootstrap iterations in MEGA5.1^64^. The full-length genomic sequences and the chromosomal attributes of the G-protein candidate genes of *Brassica* origin was retrieved from BRAD database (http://brassicadb.org/brad/). To estimate the divergence time, ClustalW was used for the pairwise alignments of coding DNA sequences of *Brassica-*specific *RGS* and *Gα* genes with their *Arabidopsis* orthologous counterparts. Ks (synonymous substitution rate) and Ka (non-synonymous substitution rate) were calculated using the DnaSP v5 program bases on the multiple sequence alignment. The divergence time (T) was calculated using the equation: T = Ks/2λ, where λ is the synonymous mutation rate, reported as 1.5 × 10^−8^ substitution per site per year for *Brassica* genes^65^.

### Gα and RGS protein-protein interaction assays

Mating based split ubiquitin system (mbSUS) was utilized to study the interaction between Gα subunit and RGS proteins^66^. Full-length CDS of *Brassica Gα* proteins were cloned in both the orientation of Nub vector (N- and C-terminal of Nub vector) and RGS proteins in Cub vector. Nub-Wt and empty Nub-vector were used as positive and negative controls, respectively and transformation and mating were performed as described^10,43^. Finally, strength and selectivity of *Brassica* Gα and RGS subunit protein interactions were determined by the growth of mated yeast cells on the selection medium lacking adenine, histidine, leucine and tryptophan (SD–AHLT), having 0, 500 and 1000 µM of methionine.

The interaction between Gα subunit and the cytosolic RGS-domain (RGS-box + C-terminal) of RGS proteins was tested using GAL4 based yeast two hybrid system. Full length CDS of *Brassica* Gα and RGS-domain were cloned into pENTR/D-TOPO entry vector. Thereafter, *Brassica* Gα proteins (bait) and RGS-domain (prey) were mobilized in pDEST-GBKT7 gateway (containing DNA binding domain) (ABRC stock: CD3-764) and pDEST-GADT7 gateway (containing having activation domain) (ABRC stock: CD3-763) vectors^67^, respectively using gateway based cloning strategy. The independent sets of bait and prey plasmids were then co-transformed into yeast strain Y2HGold. Five-six colonies were pooled and inoculated into 3 ml of liquid minimal medium deficient with leucine and tryptophan (−LT) and incubated for 16 h at 30 °C with shaking. Cultures were equalized to an OD_600_ = 0.8 and 10 μl of culture was placed on the selection medium. The interaction strength and selectivity were determined by the ability of diploid yeast cells to grow on SD–AHLT selection medium (3–5 days post inoculation), having different concentration of 3-amino-1,2,4-triazole (3-AT).

### Expression and purification of recombinant Gα and RGS proteins

The coding regions of *AtGPA1*, *BraA.Gα1* (Gα subunit of *B. rapa*), *BniB.Gα1* (Gα subunit of *B. nigra)*, *BolC.Gα1* (Gα subunit of *B. oleracea*), *BraA.RGS1box* + *Ct* and *BraA.RGS2box* + *Ct* (cytosolic RGS-domain with C-terminal region) were cloned into pET28a expression vector (Novagen, USA) and transformed into *E. coli* Rosetta-gami2 (DE3) cells (Novagen, USA). The N-terminal His-tagged recombinant Gα proteins were purified by Ni^2+^-NTA affinity chromatography^68^. Under the condition described for Gα protein purification, the recombinant RGS2-domain (RGS2box + Ct) was accumulated in the inclusion bodies (IBs). In order to maintain the purification similarities, the recombinant RGS-domain of duplicated RGS proteins were purified under denaturation condition from inclusion bodies. The pellet fraction containing the expressed protein was resuspended in extraction buffer (50 mM Tris-HCl pH 7.5; 8 M Urea; 1 mM DTT; 1 mM PMSF) and kept for 60 min at room temperature for solubilization. Solubilized protein was diluted 10 fold with extraction buffer and dialyzed overnight in wash buffer (50 mM Tris-HCl; pH 7.5; 200 mM NaCl; 1% Triton X-100 and 1 M Urea). Dialyzed protein was pooled and centrifuged at 12,000 rpm for 30 min at 4 °C and the supernatant was purified using Ni^2+^-NTA affinity chromatography similar to Gα proteins.

### G-protein activity assay of recombinant Gα and RGS proteins

*In-vitro* G-protein activity assay of the purified Gα proteins was carried out using 4,4-difluoro-4-bora-3α,4α-diaza-s-indacene-GTP Fluorophore (BODIPY-GTP FL, Invitrogen) dye in real time fluorescent assays as described previously^69^. Further to determine the GAP activity of the recombinant RGS-domain, *in-vitro* Pi release activity was also carried out using ENZchek phosphate assay kit (Invitrogen) as described previously^45^. Briefly, BraA.Gα1 protein (1 µM) was pre-loaded with GTP (1 mM) and incubated with 0.1 to 2 µM of purified RGS-domain of BraA.RGS1 and BraA.RGS2 proteins. Phosphate (Pi) released was measured as the absorbance at 360 nm using a spectrophotometer (FLUOstar Optima, BMGLab Technologies).

### Total RNA isolation, cDNA synthesis and real-time qRT–PCR

Total RNA isolation from different developmental tissues of *Brassica* species, first strand cDNA synthesis and real-time qRT-PCR were performed as described previously^43^. cDNA samples representing various growth and developmental stages were diluted 1:25 in nuclease-free water, and real-time PCRs were performed using gene-specific primers (Table S1).

### Sub-cellular localization of *B. rapa RGS* genes

In order to study the sub-cellular localization, the full-length coding regions of *BraA.RGS1* and *BraA.RGS2* were mobilized into destination binary vector pEarleyGate101 (ABRC stock CD3-683)^70^ from Gateway entry vector pENTR/D-TOPO using LR recombination strategy (Invitrogen, USA). Thereafter, stable *Arabidopsis* lines (Col-0 background) were generated independently expressing *BraA.RGS* genes fused to C-terminal YFP tag using *Agrobacterium-*mediated transformation. Hypocotyl cells of four-day old seedlings (T2 generation) grown under continuous dark conditions on 0.5 × (MS) medium without any exogenous sugar source were used to study the localization of BraA.RGS proteins. Localization of *B. rapa* RGS proteins was also tested in *Nicotiana benthamiana* epidermal leaf cells using Agro-infiltration, as described previously^71^. The overnight grown culture of *Agrobacterium* containing desired constructs were pelted down and resuspended in infiltration buffer (10 mM MgCl_2_; 10 mM MES and 100 µM Acetosyringone, pH 5.6) to an OD_600_ of 0.6. Thereafter, infiltration was carried out on the abaxial side of 4-weeks old *N. benthamiana* leaves. Infiltrated plants were kept under dark for 24 h followed by 18 h light and 6 h dark cycle for 2 days in a plant growth chamber. Fluorescence was detected using following parameters: YFP (λ ex514 nm, λ em530-560) and FM4-64 (λ ex543 nm, λ em560). Confocal images were analysed using LAS AF Lite software (Leica Microsystems). At least two independent lines were tested to establish the localization.

### Elicitor treatments

Seeds of *B. rapa* were sterilized using 0.05% HgCl_2_ and washed thoroughly using sterile distilled water. Sterile seeds were then transferred on 0.5 × (MS) medium containing 0.8% (w/v) agar and 3% (w/v) sucrose. Seeds were germinated under controlled *Brassica* growth conditions for four days under light and dark. Subsequently, uniformly grown seedlings were then adapted for 24 h in 0.5X liquid MS medium containing 1% sucrose (except for D-glucose treatment) before feeding with different phytohormones (100 µM IAA, 100 µM GA, 100 µM BAP, 100 µM ABA, 100 µM ACC and 1 µM BR); D-glucose (3%); stress and elicitors (42 °C heat, 4 °C cold, 200 mM NaCl, 200 μM MeJA, 200 μM SA, 300 μM Cu^2+^ as CuCl_2_, 500 μM Zn^2+^ as ZnCl_2_, 500 μM Mn^2+^ as MnCl_2_, 80 μM Cd^2+^ as CdCl_2_, 300 μM Pb^2+^ as PbCl_2_ and 300 μM As (V) as Na_2_HAsO_4_) each for 1, 3, 6, 12 and 24 h as described previously^41,53^. The untreated seedlings of each time points served as respective controls.

## Electronic supplementary material

Supplementary information
