# Supplementary material for: Duplicated RGS (Regulator of G-protein signaling) proteins exhibit conserved biochemical but differential transcriptional regulation of heterotrimeric G-protein signaling in Brassica species
Source: Sci Rep. 2018 Feb 1;8:2176. doi: 10.1038/s41598-018-20500-3 (PMC5794992; doi:10.1038/s41598-018-20500-3)
Supplement: Supplementary file 1 — Supplementary information [file 41598_2018_20500_MOESM1_ESM.pdf]

Title: Duplicated RGS (Regulator of G-protein signaling) proteins exhibit conserved biochemical but differential transcriptional regulation of heterotrimeric G-protein signaling in *Brassica* species

Authors: Roshan Kumar and Naveen C. Bisht

Supplementary Information

Table S1: List of primers used for amplification, protein purification, interaction and real time studies

Table S2: Inventories of *RGS* and *Gα* homologs isolated from diploid *Brassica* species.

Table S3: Nucleotide sequence identity (%) of *RGS* CDS identified from *B. rapa*, *B. nigra*, and *B. oleracea* with *Arabidopsis AtRGS1*.

Table S4: Nucleotide sequence identity (%) of *Gα* CDS identified from *B. rapa*, *B. nigra* and *B. oleracea* with *Arabidopsis AtGPA1.*

Table S5: The synonymous base substitution (Ks) and divergence time estimation of *B. rapa*, *B. nigra* and *B. oleracea RGS* and *Gα* genes with corresponding *Arabidopsis* ortholog.

Table S6: Summary of *RGS* and *Gα* genes from three diploid species of *Brassica* 'U' triangle.

Table S7:- Summary of gene content in genomic blocks 'I' and 'L' shared between *A. thaliana* and three sub-genomes of *B. rapa* and *B. oleracea.*

Fig. S1: Nucleotide sequence alignment of *Brassica RGS* coding sequences. The sequence alignment of CDS of *RGS* genes from *B. rapa*, *B. nigra* and *B. oleracea* with *Arabidopsis AtRGS1* was performed using ClustalW (http://www.clustal.org).

Fig. S2: Nucleotide sequence alignment of *Brassica Gα* coding sequences.

Fig. S3: Interaction analysis between cytosolic RGS domain (RGS box + Ct) of *Brassica* RGS and Gα proteins using yeast two hybrid assay.

Fig. S4: Expression and purification of recombinant Gα and RGS-domain proteins.

Fig. S5: GTPase activity assay of RGS-domain proteins on Gα proteins.

Fig. S6: Sub-cellular localization of BraA.RGS1 and BraA.RGS2 proteins.

Table S1: List of primers used for amplification, protein purification, interaction and real time studies

	**Sequence (5'-3')**
**Gene amplification primers**	
RGS1_Dtopo FP	CACCATGGCGAGTGGATGYGCTMWAC
RGS1_Dtopo RP	TTAACCGGGACTASTGCATCTGGA
RGS2_Dtopo FP	CACCATGGCGAGTGGATGTGCTAAACG
RGS2_Dtopo RP	TTAACTAGGACTGCTATATCTAGA
RGS box+ Ct (cons) Dtopo FP	CACCCCTCTTCTCTCACAAATCAGC
BraA.RGS2 box+ Ct Dtopo FP	CACCCCTCTTCTCGCACAAATCAGC
**pET28a cloning primers**	
pET28a_Gα1 FP	ATATATCATATGGGCTTACTCTGC
pET28a_Gα1 RP	ATATATGAATTCTCATAAAAGGCCAGC
pET28a_BraRGS1box+Ct FP	ATTAGCTAGCCCTCTNCTYTCACARATCAGC
pET28a_BraRGS1box+Ct RP	ATTAGAATTCTTAACCGGGACTAGTGCATCT
pET28a_BraRGS2box+Ct FP	ATTAGCTAGCCCTCTACTCGCACAAATCAGC
pET28a_BraRGS2box+Ct RP	ATTAGAATTCTTAACTAGGACTGCTATATCT
**Split ubiquitin primers**	
Gα1_SUS FP	ACAAGTTTGTACAAAAAAGCAGGCTCTCAACCACCATGGGCTTACTCTGCAGT
Gα1_SUS RP	TCCGCCACCACCAACCACTTTGTACAAGAAAGCTGGGTATAAAAGGCCAGCYTCCAA
RGS1-2_SUS FP	ACAAGTTTGTACAAAAAAGCAGGCTCTCCAACCACCATGGCGAGTGGATG
RGS1_SUS RP	TCCGCCACCACCAACCACTTTGTACAAGAAAGCTGGGTATATACCGGGACTAGTGCA
RGS2_SUS RP	TCCGCCACCACCAACCACTTTGTACAAGAAAGCTGGGTATATACTAGGACTGCTATATCTAGA
**Real-time primers**	
BraA.RGS1 FP	GATACCTGATAGTGGTTTG
BraA.RGS1 RP	CCTTATCGAATCACCTTCAGGT
BraA.RGS2 FP	GCTTCAAGAACAGACAA
BraA.RGS2 RP	CGCGAACTCCATAAGCGACTTT

Table S2: Inventories of *RGS* and *Gα* homologs isolated from diploid *Brassica* species.

***Brassica* species**	***RGS* and *Gα* homologs**	**CDS (bp)**	**Protein (amino acids)**	**Amino acid identity (%) with *Arabidopsis* ortholog**	**Molecular weight (kDa)**
***B. rapa* (*A genome*)**	*BraA.RGS1*	1377	458	89.1	52.3
	BraA.RGS2	1368	455	83.8	52.8
	BraA.Gα1[10]	1152	383	96.9	44.5
***B. nigra* (*B genome*)**	*BniB.RGS1*	1386	461	89.1	52.8
	BniB.RGS2	1374	457	85.4	52.4
	BniB.Gα1[43]	1152	383	96.9	44.4
***B. oleracea* (*C genome*)**	*BolC.RGS1*	1377	458	89.3	52.5
	BolC.RGS2	1368	455	84.9	52.2
	BolC.Gα1	1152	383	96.4	44.5

[10]Arya et al. 2014; [43]Kumar et al. 2014

Table S3: Nucleotide sequence identity (%) of *RGS* CDS identified from *B. rapa*, *B. nigra*, and *B. oleracea* with *Arabidopsis AtRGS1*.

	BraA.RGS1	***BniB.RGS1***	***BolC.RGS1***	***BraA.RGS2***	***BniB.RGS2***	***BolC.RGS2***
AtRGS1	88.4	89.1	88.4	87.6	87.9	88.3
BraA.RGS1	***	93.2	97.1	88.5	88.6	88.6
BniB.RGS1		***	93.3	88.3	88.8	88.7
BolC.RGS1			***	88.5	88.6	88.9
BraA.RGS2				***	93.4	98.2
BniB.RGS2					***	93.7
BolC.RGS2						***

Table S4: Nucleotide sequence identity (%) of *Gα* CDS identified from *B. rapa*, *B. nigra* and *B. oleracea* with *Arabidopsis AtGPA1*.

	BraA.Gα1	***BniB.Gα1***	***BolC.Gα1***
AtGPA1	91.7	92.2	91.8
BraA.Gα1	***	97.3	98.1
BniB.Gα1		***	97.1
BolC.Gα1			***

Table S5: The synonymous base substitution (Ks) and divergence time estimation of *B. rapa*, *B. nigra* and *B. oleracea RGS* and *Gα* genes with corresponding *Arabidopsis* ortholog.

**Gene pair**	**Ks**	**Ka**	**Ka/ks**	**Divergence time (mya)**
***A. thaliana* and *B. rapa* (A genome)**				
AtRGS1-BraA.RGS1	0.3669	0.0726	0.1978	12.23
AtRGS1-BraA.RGS2	0.4192	0.0515	0.1228	13.97
AtGPA1-BraA.Gα1	0.4269	0.0158	0.0370	14.23
***A. thaliana* and *B. nigra* (B genome)**				
AtRGS1-BniB.RGS1	0.3839	0.0671	0.1747	12.79
AtRGS1-BniB.RGS2	0.4705	0.0534	0.1134	15.68
AtGPA1-BniB.Gα1	0.3923	0.0146	0.0372	13.07
***A. thaliana* and *B. oleracea* (C genome)**				
AtRGS1-BolC.RGS1	0.3487	0.0683	0.1958	11.62
AtRGS1-BolC.RGS2	0.4276	0.0500	0.1169	14.25
AtGPA1-BolC.Gα1	0.3995	0.0180	0.0450	13.30

Table S6: Summary of *RGS* and *Gα* genes from three diploid species of *Brassica* 'U' triangle.

***Brassica* species**	***RGS* and *Gα* genes**	**Gene ID***	**Gene size (bp)***	**Linkage group***	**No. of exons**	**No. of introns**
B. rapa* *(A)	*BraA.RGS1*	Bra025181	2303	A06	10	9
	BraA.RGS2	Bra017336	2911	A09	10	9
	BraA.Gα1	Bra007761	2425	A09	13	12
B. nigra* *(B)	*BniB.RGS1*	BniB025334	2417	B08	10	9
	BniB.RGS2	BniB022140	2253	scaffold_28.1	10	9
	BniB.Gα1	BniB042187	2591	scaffold_683.1	13	12
B. oleracea* *(C)	*BolC.RGS1*	Bol042781	2364	C07	10	9
	BolC.RGS2	Bol032605	2909	C09	10	9
	BolC.Gα1	Bol044641	2419	C08	13	12

*Obtained from BRAD database (http://brassicadb.org/brad/index.php); Bolbase (http://ocri-genomics.org/bolbase/)

Table S7:- Summary of gene content in genomic blocks 'I' and 'L' shared between *A. thaliana* and three sub-genomes of *B. rapa* and *B. oleracea* (http://www.brassicadb.org/). The sub-genomes of *B. rapa* and *B. oleracea* are LF (least gene fractionized), MF1 (moderately gene fractionized) and MF2 (most gene fractionized).

	A. thaliana	***B. rapa* (A genome)**			***B. oleracea* (C genome)**		
		**LF**	**MF1**	**MF2**	**LF**	**MF1**	**MF2**
**Number of shared genes in genomic block 'I'**	786	357	355	81	340	370	83
**Number of *Brassica* lineage specific genes in genomic block 'I'**		98	119	26	47	20	20
**Percentage (%) of syntenic *Arabidopsis* genes retained in *Brassica* species in genomic block 'I'**		45.4	45.1	10.3	47.0	47.0	10.5
**Number of shared genes in genomic block 'L'**	529	225	173	115	223	157	105
**Number of *Brassica* lineage specific genes in genomic block 'L'**		30	71	22	17	18	11
**Percentage (%) of syntenic Arabidopsis genes retained in *Brassica* species in genomic block 'L'**		42.5	32.7	21.7	42.1	29.6	19.8

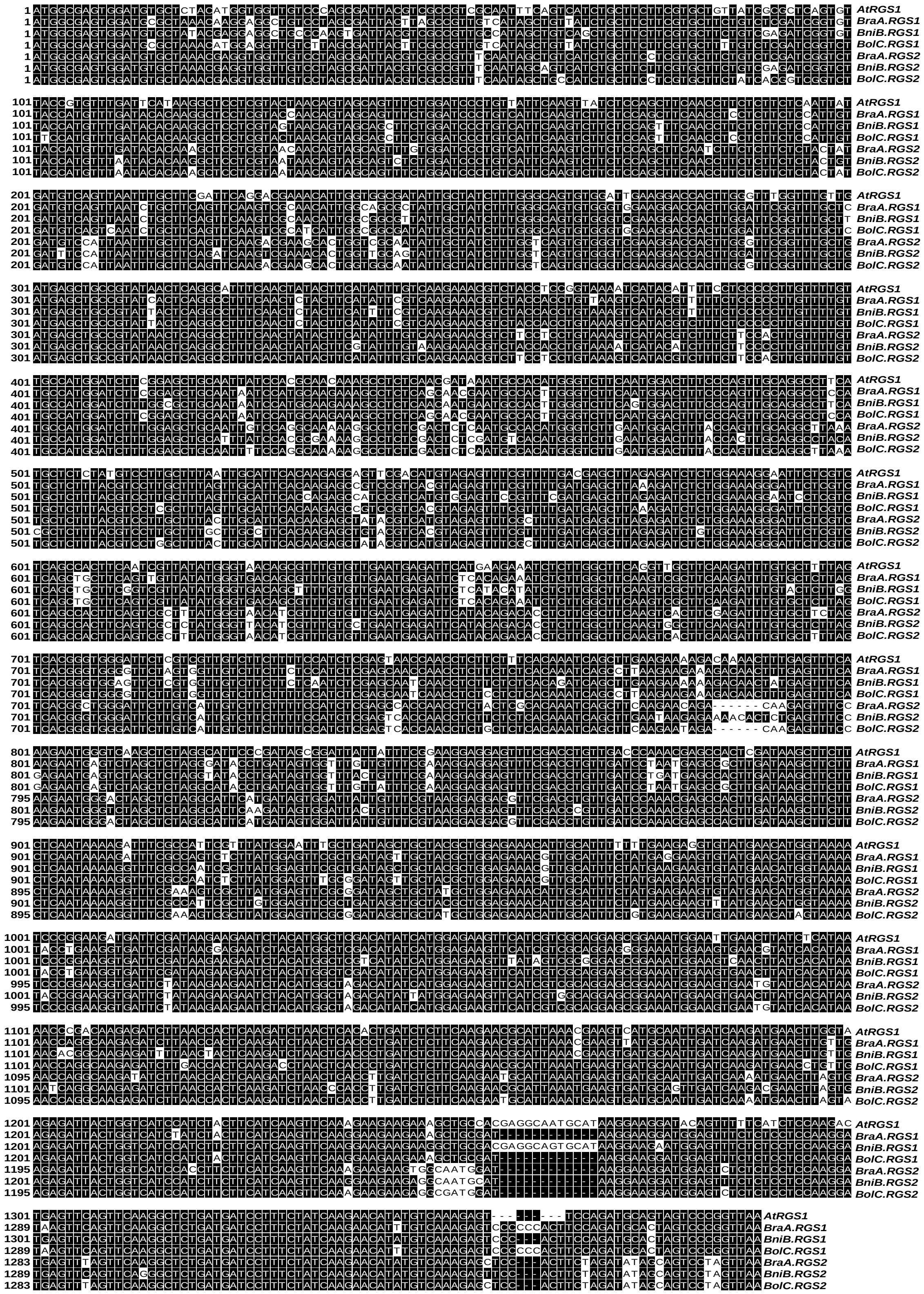

Fig. S1: Nucleotide sequence alignment of *Brassica RGS* coding sequences. The sequence alignment of CDS of *RGS* genes from *B. rapa*, *B. nigra* and *B. oleracea* with *Arabidopsis AtRGS1* was performed using ClustalW (http://www.clustal.org).

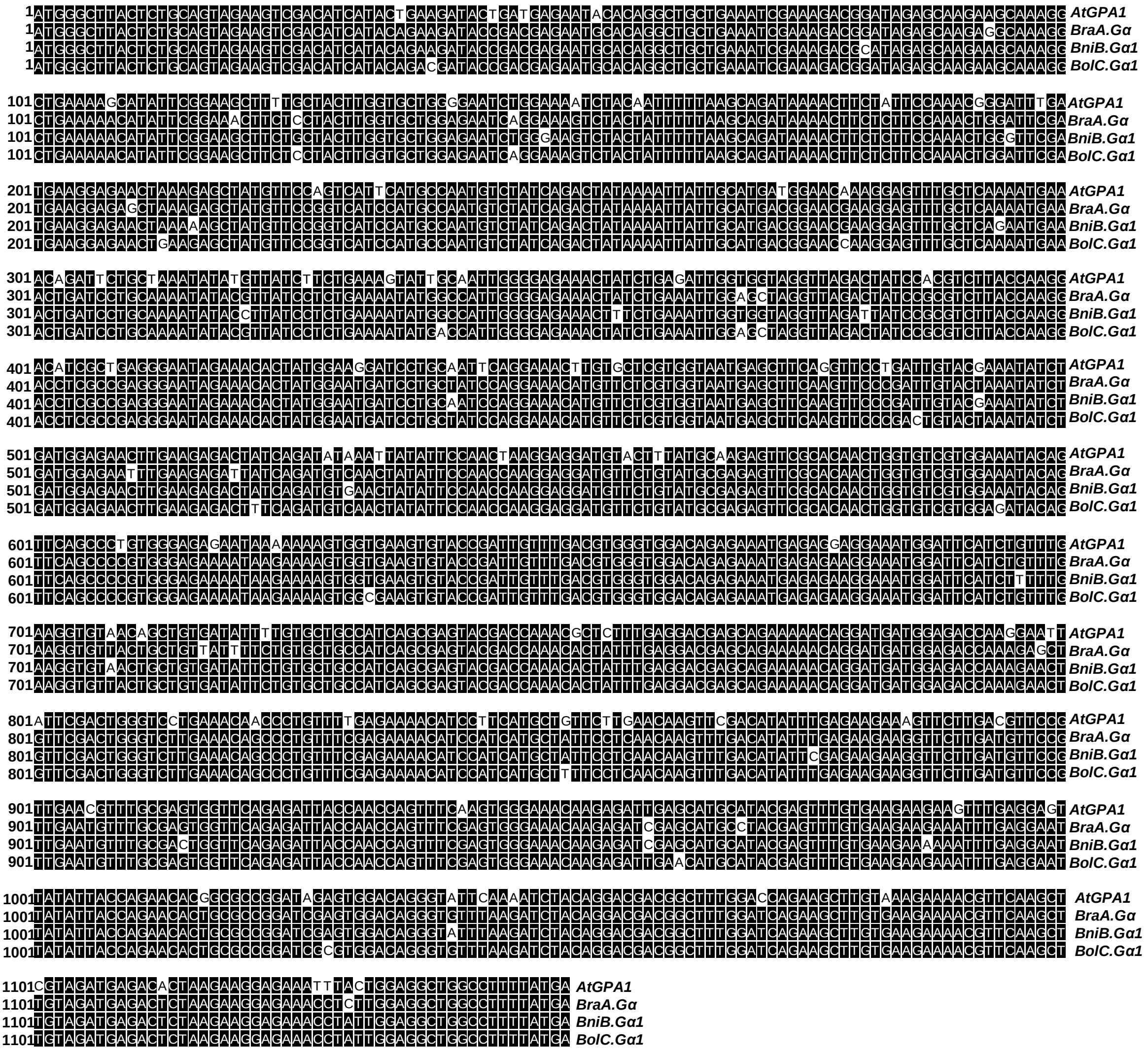

Fig. S2: Nucleotide sequence alignment of *Brassica Gα* coding sequences. The sequence alignment of CDS of *Gα* subunit genes from *B. rapa*, *B. nigra* and *B. oleracea Arabidopsis AtRGS1* was performed using ClustalW (http://www.clustal.org).

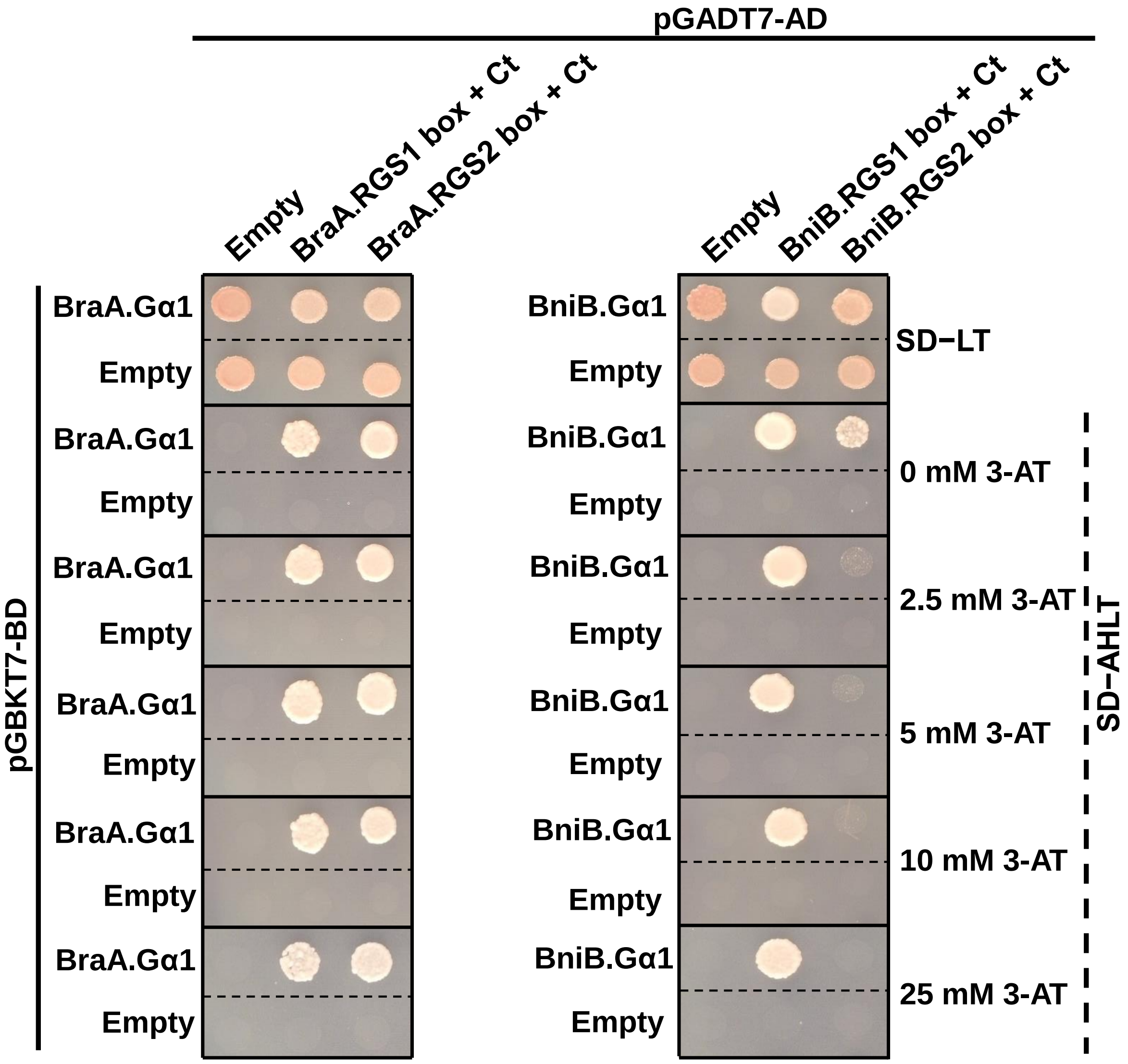

Fig. S3: Interaction analysis between cytosolic RGS-domain of *Brassica* RGS and Gα proteins using yeast two hybrid assay. The interaction strength and selectivity of *B. rapa and B. nigra* RGS domain protein (in pGADT7-AD) with their cognate Gα proteins (in pGBKT7-BD) were determined by growth based assay of diploid yeast cells on the minimal medium lacking adenine, histidine, leucine and tryptophan (SD-AHLT) containing different concentration (0, 2.5, 5, 10 and 25 mM) of the 3-AT (3-amino-1.2.4-triazole). Two independent biological replicates of the experiment were carried out with similar results.

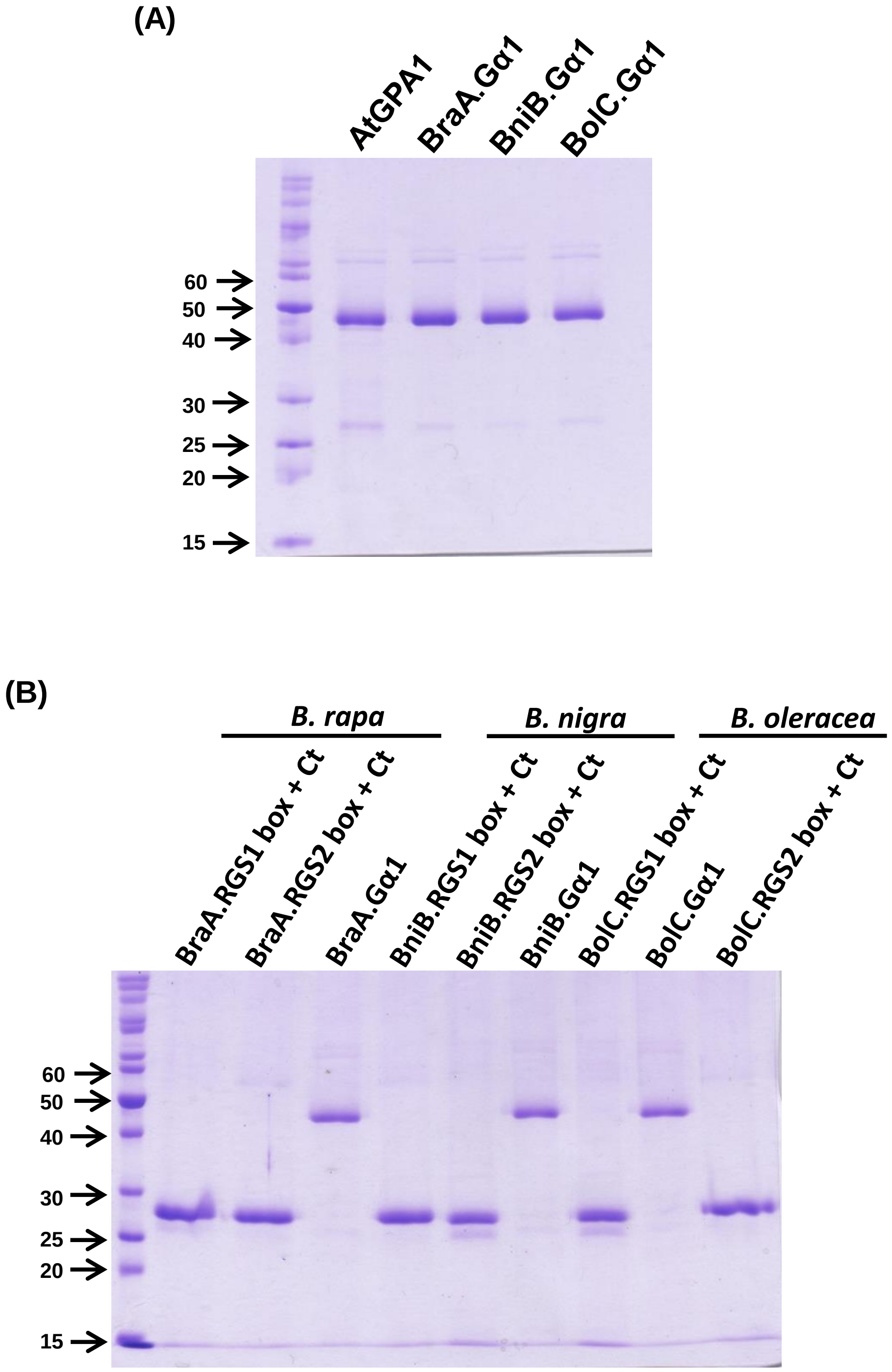

Fig. S4: Expression and purification of recombinant Gα and RGS-domain proteins. (A) SDS-PAGE analysis of purified recombinant BraA.Gα1 (*B. rapa*), BniB.Gα1 (*B. nigra*), BolC.Gα1 (*B. oleracea*) and AtGPA1 (*A. thaliana*) proteins. (B) SDS-PAGE analysis of purified recombinant 'RGS-domain' of RGS proteins of three *Brassica* species (*B. rapa*, *B. nigra* and *B. oleracea*) with their Gα protein.

(A)

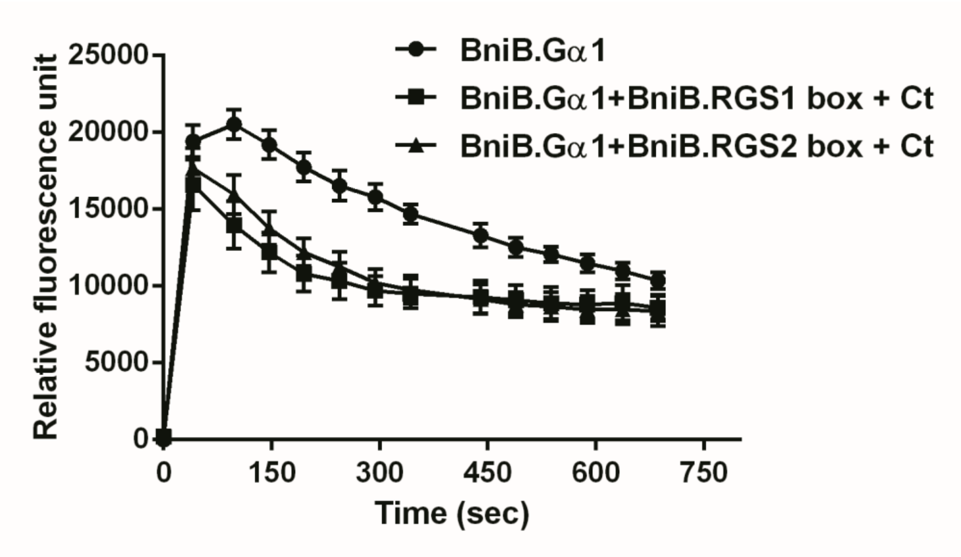

(B)

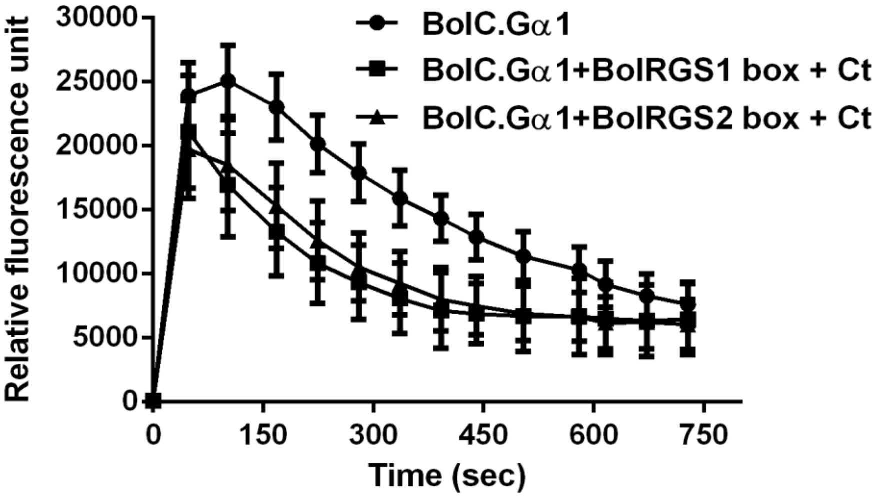

Fig. S5: GTPase activity assay of RGS-domain proteins on Gα proteins. Effect of (A) BniB.RGS1/2 domain on GTP-hydrolysis of BniB.Gα1, and (B) BolC.RGS1/2 proteins on GTP-hydrolysis of BolC.Gα1, using BODIPY fluorescent dye in real-time fluorescence assays. Experiments were carried out three times and data was averaged (± S.E). Data was analysed using GraphPad Prism version 6.0.

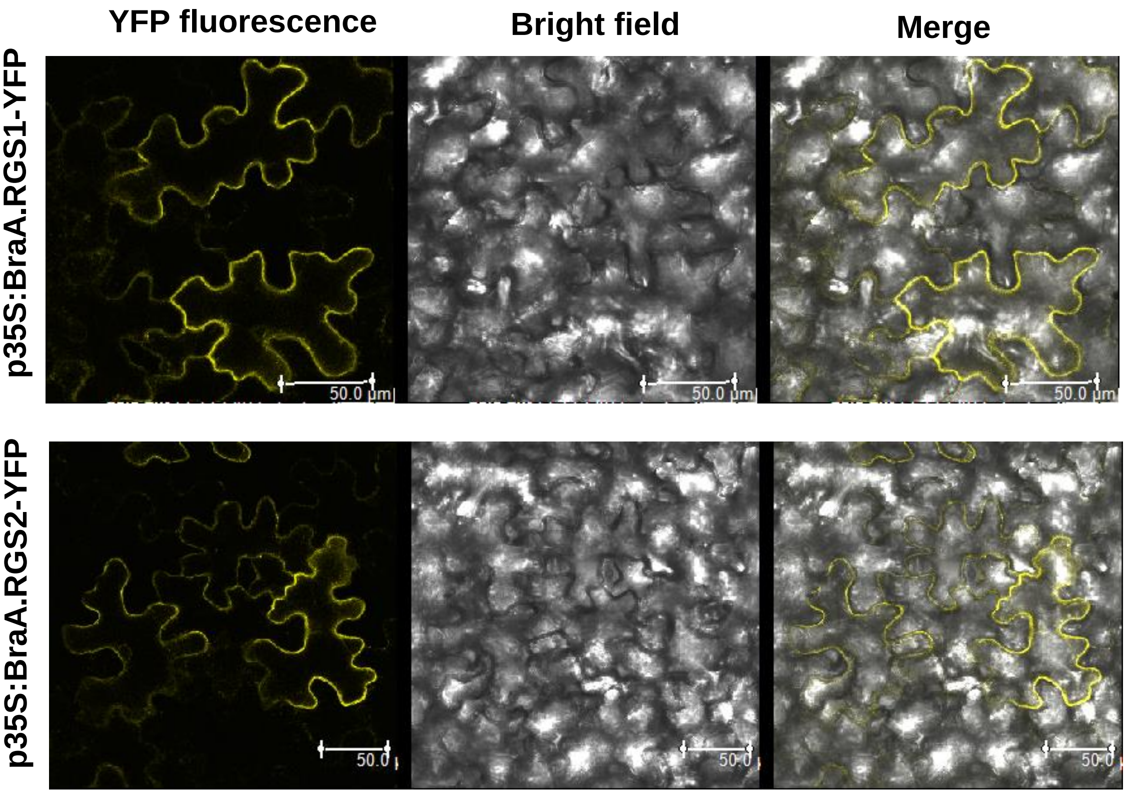

Fig. S6: Sub-cellular localization of BraA.RGS1 and BraA.RGS2 proteins. Localization was studied in transiently transformed by *N. benthamiana* leaves by Agro-infiltration.
